# Evaluation of water quality index and geochemical characteristics of surfacewater from Tawang India

**DOI:** 10.1038/s41598-022-14760-3

**Published:** 2022-07-09

**Authors:** Nisha Gaur, Arpan Sarkar, Dhiraj Dutta, B. J. Gogoi, Rama Dubey, Sanjai Kumar Dwivedi

**Affiliations:** Defence Research Laboratory, Strategic Product Development Division, DRDO, Tezpur, Assam India

**Keywords:** Biogeochemistry, Environmental sciences, Hydrology

## Abstract

In this study,the water samples were collected from 31 sites of Tawang, Arunachal Pradesh, India (North-Eastern Himalaya), during the winter season to check the suitability of water for drinking and irrigation purposes.The study scientifically demonstrates the estimation of Water quality index (WQI) andhydrogeochemical characteristics of surface water samples by utilizing multivariate statistical methods. The main water quality parameters considered for this study were TDS, conductivity, salinity, pH, hardness, cations and anions. WQI was calculated in order to find out the deviation in the water quality parameters particularly with respect to BIS permissible limits.The major influencing factors responsible for the variation in these parameters were derived by using Principal component analysis (PCA) and Correlation matrix.To check the suitability of water for drinking purpose, hydrogeochemical facies and rock water interaction was derived by using well established methods such as Piper Plot (determine water type), WQI (Quality monitoring), and saturation index (for mineral dissolution). The results revealed that the silicate weathering was the main ionic source in comparison to carbonate weathering which is due to the higher dissolution capacity of silicate minerals.The results of the scattered plot between (Ca^2+^ + Mg^2+^)–(HCO_3_ˉ + SO_4_^2^ˉ) versus (Na^+^ + K^+^)–Clˉ (meq/L) highlighted thation exchange occurs between Mg^2+^ and Ca^2+^ofsurface water with Na^+^ and K^+^of rock /soil. This means that calcium ion was getting adsorbed, and sodium ion was getting released. The Ca^2+^–Mg^2+^–HCO_3_ˉ, Na^+^–HCO_3_ˉand Na^+^–Clˉ type of surface water suggested permanent and temporary hardness respectively in the studied region. The dominant cations of this study were Na^+^ and Ca^2+^ while the dominant anions were HCO_3_ˉ and SO_4_^2^ˉ. In order to check the suitability of water sources for irrigation, parameters like, Magnesium hazard (MH), Total hardness (TH), Permeability Index (PI), Kelly Index (KI), Sodium adsorption rate (SAR), Sodium percentage (Na%), and Residual sodium carbonate (RSC) were determined. The results showed that 93% of the samples had PI score < 75, which indicates the suitability of the water for irrigation. Also the WQI calculation showed an average WQI value of 82.49, amongst which 61% samples were in the range of 0–50 being considered as good for drinking, while 39% were catageorised as unsuitable for drinking showing a value of > 50. Hence the above findings reveal that geogenic activities play a major role in influencing the water quality of Tawang region. Hence suitable water treatment technologies or methods might be used to eliminate thenon desirable elements and minerals present in surface water.

## Introduction

For all life forms present on earth water is a basic need fulfilled from variousnatural resources. It is desired that, the water being consumed should not contain any microbes or harmful chemicals thatcan cause damage to life. Due to industrialization, surface and groundwater has been contaminated with a widevariety of pollutants.Both natural (precipitation, the geography of the watershed, atmosphere, and geology) and anthropogenic (industrial activities, domestic and/or agricultural run-off) activities determine the chemical, physical and biological composition of surface and groundwater. Water contamination leads to deterioration of water quality whichthreatens the life present on earth as well as disturbs theeconomic advancement and social success^1,2^.

Tawang, a small district in Arunachal Pradesh, Indialocated at an altitude of 4500 m is surrounded with high mountains, glaciers and lakes. The water requirement of local population is met from the surface water sources such as springs, lakes, ponds and rivers. At the mentioned altitude, the only propable sources of contamination are natural and geogenic activities. Few reports onwater quality of Eastern Himalayan region showed severe fecal contamination of surface water sources and the reservoir tank used by the community, leading to high health risk^3,4^. Additionally, the geological composition of sourrounding soil and rock-formation strongly affects the water quality, since the flowing and stagnant water comes in their close contact.Now a days hydrogeochemical studies are gaining momentum worldwide to ascertain the impact of geological strata on water quality of natural water bodies. The studies throw light on water rock interaction leading to presence of certain minerals in the water mainly derived from weathering of rocks and dissolution of minerals. The reported studies have highlighted the presence of silicates, carbonates, alkali and alkaline earth metals, heavy metals etc. based on the hydrogeochemical analysis of water sources by using different statistical approaches^5^. Hence the hydrogeochemical characterization of surface water demonstrates thelevel of minerals and ions contributing to aquifer's composition. Furthermore, the surface water is also susceptible to other forms of contamination arising from domestic activities. The constant decline of surface water quality not only affects the humans but also poses a serious threat to the ecosystems flourishing within it. Hence, to maintain the health of any water source, certain water quality indicators or parameters must be monitored regularly to keep the aquifer system performing at its best^6,7^. Regular monitoring of water quality is a crucial part for determining the baseline data which would help in identifying any existing problems, or any issues that could emerge in the future related to water quality.

Water quality is generally defined by its physicalapperance (colour, odour, taste), chemical (pH, turbidity, hardness, alkalinity, total solids, presence of metallic or non-metallic salts) and biological properties.Many researchers have proposed WQI in the form of a simple expression in order to represent the general quality of surface water as there are a variety of physical, chemical and biological water quality parameters^8–10^. It is a concise and comprehensive method to express the quality of water for different stages of usage and is commonly represented by a single number. WQI of any water sample is calculated by aggregating the values of different parameters that givesa single number which expresses the quality or contamination status of water. WQI not only can be used for the prediction of pollutants present in water but also compares the water qualities of different sources and hence decides the proper usage of water resources^11,12^. It is very difficult to comprehend a complex and large data matrix comprising a large number of parameters while calculating WQI. To overcome this problem and make the process less subjective, different multivariate techniques (Principal Component Analysis (PCA), factor analysis, etc.) helps in better interpretation of the results. Several parameters are monitored and analyzed simultaneously along with studying environmental issues. Ideally, PCA can reduce the dimensionality of the multivariate data set while maintaining its structure to the maximum extent. Hence, while dealing with environmental data PCA has often been used^13^.

In recent years Geographic Information System (GIS) technique is emerging as a powerful tool for storing, assessing, monitoring and displaying spatial dataof surface and groundwater quality. This tool is also effective in developing solution for water resource related problems, understanding the natural environmentand managing water resources on required scale.Additionally, for evaluation and analysis of spatial information of water resources, Inverse Distance Weighted (IDW) interpolation methodalong with GIS techniques has proved as a powerful tool. For transforming huge data sets to generate various spatial distribution maps and projections revealing trends, associations, and sources of contaminant it is an economically feasible and time-efficient technique^14–16^.

Therefore, in the present study, the physico-chemical and bacterial properties of surface water samples collected from different sites of Tawang were determined and compared with other related studies. Main emphasis was laid on interaction between physico-chemical parameters of water by using Principal component analysis (PCA) and correlation matrix. The selected parameters were normalized by using a z-score before PCA as it requires individual indicators to have a common unit. In the final step, the Pearson correlation analysis was done followed by water quality index calculation. These statistical analysis tools are important to understand the vital variables which affect the quality of water. For spatial evaluation of various surface water quality parameters, the GIS technique has been used.

Furthermore, the hydrogeochemical facies and hydrogeochemical signatures such as ion exchange process, rock-water interaction, and dissolution were also studied for the analysis of chemical characteristics of surface water. Conventional graphical plots such as Magnesium hazard (MH), Total hardness (TH), Permeability Index (PI), Kelly Index (KI), Sodium adsorption rate (SAR), sodium percentage (Na%), and residual sodium carbonate (RSC) have been used to determine the various hydrogeochemical processes controlling the hydrochemical characteristics of water collected from the study area. Hence this study will throw some light on the untouched surface water sources in the Tawang area and thus can act as a baseline for water quality assessment in various other districts of Arunachal Pradesh and Northeastern region of India.The ultimate objective of the present study was to highlight the interaction between geogenic factors and water and correlate it with the water quality index, so that its suitabilitycan be ascertained for potability and irrigation purpose.

## Study area

**Figure 1:**
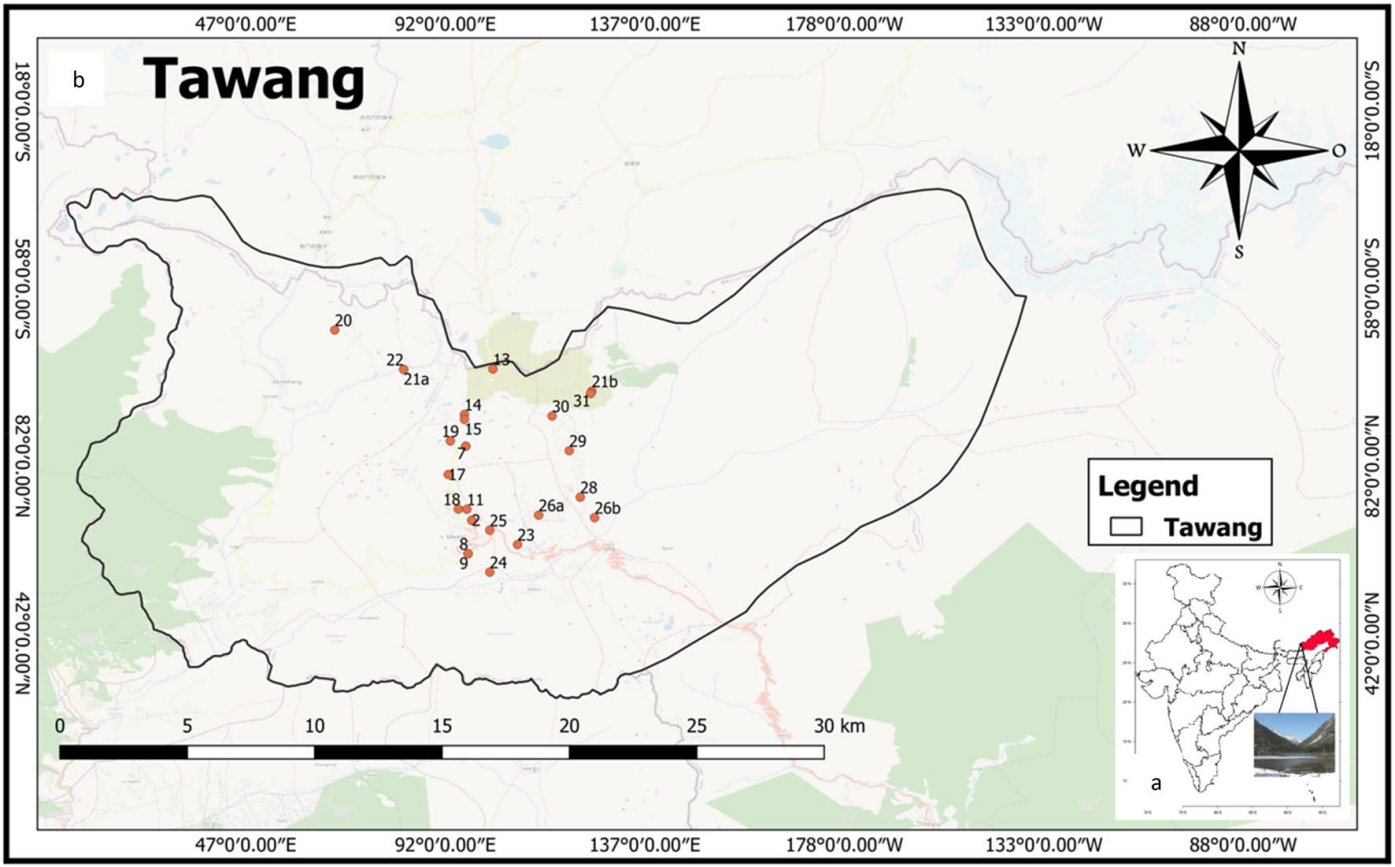
Location Map of Ecological hotspot of Tawang, Arunachal Pradesh, India. (**a**) Location of Tawang on Indian map (**b**) Sample collection sites. QGIS Software Desktop 3.18.3 (https://qgis.org/en/site/forusers/download.html).

The Ecological hotspot of Tawangis in the Arunachal Pradesh (latitude 91° 33’ E to 92° 26’E; longitude 27° 29’N to 27°52’N) at an altitude of 1800–3300 m above mean sea level in Eastern Himalayan Region (Fig. 1a and b). This place is famous for its 400-year-old biggest Buddhist monastery and important pilgrim center for the followers of Buddhism. It is also famous for its natural beauty which enchants the traveller. It was observed that the maximum number of tourists visit this place during the summer season due to pleasant weather. The people of the forward location of Tawang depend on natural water sources for their daily needs. Because of the tough terrain, it is very difficult to bring fresh water from other locations so there is a need to preserve these water bodies.

During winters and the rainy season (because of landslides and heavy snowfall), the survival condition is very harsh and very difficult for local people to collect potable water from a nearby area. Therefore, this area was selected to check the water quality from different natural water sites of Tawang. As the population of this area is comparatively less and no anthropogenic activity was observed so thirty-one different natural freshwater sites alongthe forward location of Tawang, Arunachal Pradesh were chosen for sample collection to ascertain thesutability of water quality for drinking and irrigation.

## Methodology

The water sampling from 31different locations of Tawang district, Arunachal Pradesh was done during the winter season (December 2020) as per the standard procedures of the American Public Health Association^17^. For the collection and analysis of various water variables, standard methods were followed^17,18^. With the help of a global positioning system (GPS) as shown in Fig. 1b, the sampling locations were marked. All the plastic bottles were thoroughly washed and dried before sample collection and the bottles were rinsed with water sample to be collected at the time of collection. After sample collection proper labelling was done. The latitude, longitude, and altitude of all the sampling sites along with the source were recorded during the sample collection using a GPS system (Model: Garmin GPS 72H) as mentioned in Table 1.

**Table 1:** Details of sampling site.

Location	Latitude	Longitude	Altitude (meter)
1	272°05´94.17´´	905°61´39.43´´	2347
2	27°36´02.73´´	091°52´33.82´´	2793
3	27°36´02.73´´	091°52´33.82´´	2793
4	27°36´02.46´´	091°52´33.69´´	2713
5	27°39´34.26´´	091°52´16.43´´	2511
6	27°39´34.26´´	091°52´16.43´´	2511
7	27°39´34.26´´	091°52´16.43´´	2511
8	27°34´27.24´´	091°52´23.22´´	2539
9	27°34´27.23´´	091°52´23.33´´	3080
10	27°34´27.23´´	091°52´23.33´´	3080
11	27°36´34.35´´	091°52´19.52´´	4350
13	27°43´14.49´´	091°53´34.06´´	4465
14	27°41´04.70´´	091°52´12.27´´	4348
15	27°40´50.87´´	091°52´12.67´´	4252
16	27°50´.88´´	091°52´12.74´´	4303
17	27°38´13.42´´	091°51´26.30´´	3895
18	27°36´34.70´´	091°51´55.09´´	4056
19	27°39´49.29´´	091°51´32.36´´	3782
20	27°45´06.06´´	091°46´02.07´´	4219
21A	27°43´13.40´´	091°49´19.11´´	2699
21B	27°42.190´	091°58.274´	4077
22	27°43´13.47´´	091°49´18.98´´	2401
23	27°34´53.37´´	091°54´44.24´´	2641
24	27°33´34.41´´	091°53´24.96´´	2474
25	27°35´34.41´´	091°53´24.96´´	2262
26A	27°36´17.4´´	091°55´44.7´´	4048
26B	27°36.173´	091°58.409´	2856
28	27°37.146´	091°57.723´	3162
29	27°39.358´	091°57.200´	3791
30	27°41.008´	091°56.385´	4048
31	27°42.075´	091°58.217´	4077

### Physiochemical parameters determination

**Figure 2:**
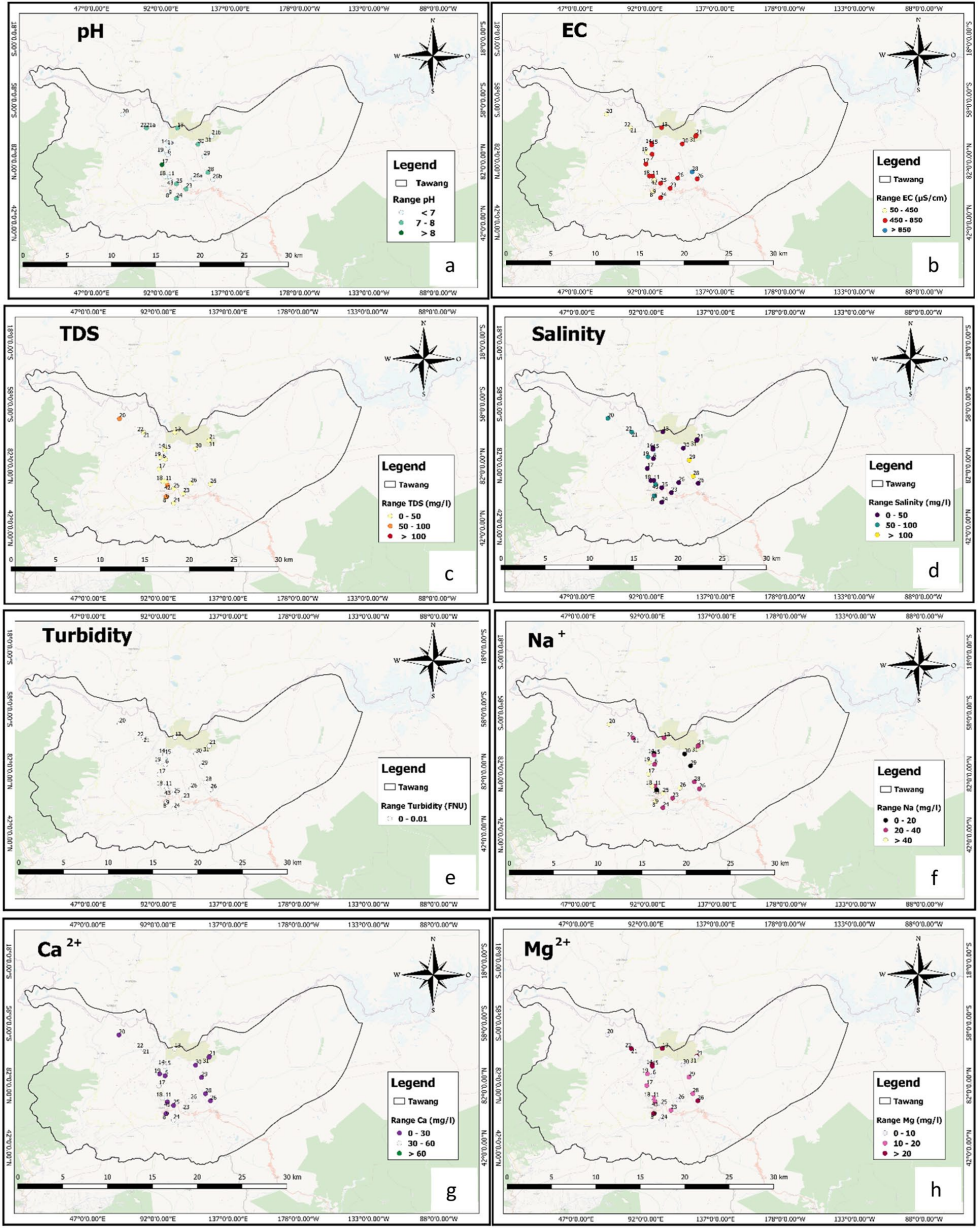

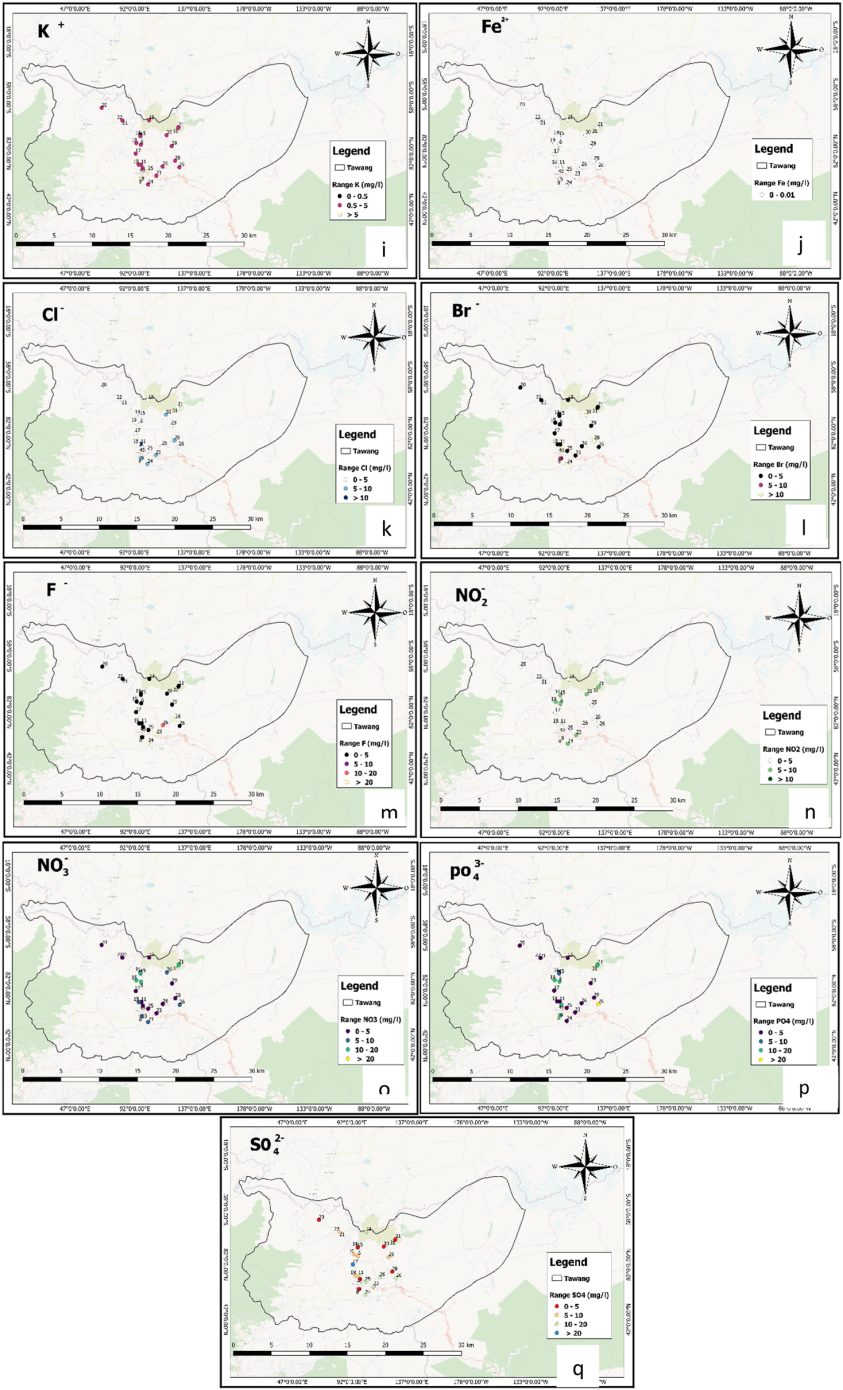
Spatial distribution map of (**a**) pH, (**b**) EC, (**c**) TDS, (**d**) Salinity, (**e**) Turbidity, (**f**) Sodium (Na^+^), (**g**) Calcium (Ca^2+^) and (**h**) Magnesium (Mg^2+^). Spatial distribution map of (**i**) Potassium (K^+^), (**j**) Iron (Fe^2+^), (**k**) Chloride (Cl^-^), (**l**) Bromide (Br^-^), (**m**) Fluoride (F^-^), (**n**) Nitrite (NO_2_^-^), (**o**) Nitrate (NO_3_^-^), (**p**) Phosphate (PO_4_^3-^) and (**q**) Sulphate (SO_4_^2-^) QGIS Software Desktop 3.18.3 (https://qgis.org/en/site/forusers/download.html).

After sample collection, the bottles were taken to the laboratory in an icebox to avoid any unusual change in water quality and stored at 4 °C for further analysis. Electrical conductivity, TDS, and salinity were analyzed with the help of EUTECH Instruments CD650 (Thermo Scientific, United State). A digital pH meter (EUTECH pH 610) was used to estimate pH. Turbidity meter (EUTECH TN 100) was used to determine turbidity and iron was estimated by using a UV–Vis spectrophotometer (Analytikjena SPECORD 205). The acid titration method was carried out for Bicarbonate analysis. Chloride, Nitrite, Flouride, Nitrate, and Sulfate were evaluated by using Ion Chromatography (Metrohm, 882 Compact IC plus, 858 Professional Sample Processor). Sodium, Potassium, Calcium, and Magnesium were analyzed by following the standard methods specified in IS-1500:2012^18,19^. All the analysis was done as per the mathods methods mentioned in APHA^20^.The Physiochemical properties and spatial distribution pattern of collected water samplesare shown in Fig. 2a–q.

### Quality assurance and quality control (QA/QC)

Standard solutions were used for the pre-calibration of all the instruments used for all the analysis as per the company guidelines to maintain the accuracy and precision in observations. Before each in situ observation all the electrodes were properly washed with double distilled water. Conditioning of probes should be done in the sample before each use for best stabilization time. Freshely prepared buffer solutions of two different units were used for calibration of rinsed and dry pH probe. For each chemical analysis, a blank sample had been run for each chemical analysis and for each quality parameter at least three observations had been taken. Standard operating protocols were adopted for each instrument and chemical analysis with adequate safety throughout the study period. To get the accurate data/information the analytical grade chemicals and glasswares (Borocile, Merk Thermo Fishers) were used for chemical analysis. To get the linear calibration curves in Ion chromatography, the calibration was performed by running the replicate standards of different anions.

### Multivariate statistic methods

Kolmogorov–Smirnov (K–S) test with the SPSS 21.0 Pro software packages (SPSS Ins., Chicago., USA) (https://www.ibm.com/support/pages/downloading-ibm-spss-statistics-21) was used to assess the normality of the water quality parameters. To compare the spatial and temporal variations of 15 water quality parameters measured in different sites of Tawang, descriptive statistical analysis including one-way Analysis of Variance (ANOVA) was performed. Tukey’s multiple range tests were performed to evaluate the large differences in mean values of water quality parameters. The Pearson’s correlation coefficient (r) was calculated to determine the correlation between variables. Principal Component Analysis (PCA)was used to analyze the spatial and temporal changes in water quality. In addition, PCA also extracts the pollution factor and recognizes pollution sources applied in water quality analysis. PCA also reduces the data sets and enables the formation of new factors. In this case, the parameters have been reduced from 15 to 13. The classification of factor loading is strong (> 0.75), moderate (0.75–0.50), and weak (0.50–0.30) relating to the absolute loading value. To consider the suitability of the data to run PCA, Kaiser–Meyer–Olkin (KMO) test was applied and if the value of the KMO test is below 0.5 it is unacceptable, between 0.5 to 0.7 is sufficient, and > 0.7 is considered as good. PAST Software 4.03 (https://www.softpedia.com/get/Science-CAD/PAST.shtml) was used for correlation analysis and principle component analysis. These analyses were applied as a complementary tool to describe water quality deterioration^8,9,21,22^.

### Water quality index (WQI)

Several national and international organizations have formulated a huge number of water indices such as Weight Arithmetic Water Quality Index (WAWQI), National Sanitation Foundation Water Quality Index (NSFWQI), Canadian Council of Ministers of the Environment Water Quality Index (CCMEWQI), Oregon Water indices which are followed worldwide^23,24^.

Water quality index (WQI) is defined as “the grading technique that provides the combined effect of each of the water quality parameters on the overall water quality for human consumption”^25^. It is widely used to characterize the availability of potable water resources and their usefulness for domestic purposes. In the present study the weight arithmetic water quality index was determined as per the methods mentioned in the literature^26^. The mean values of analyzed 15 parameters (pH, turbidity, EC, TDS, Salinity, Clˉ, Brˉ, NO_2_ˉ, NO_3_ˉ, PO_4_^3^ˉ, SO_4_^2^ˉ, Na^+^, Ca^2+^, Mg^2+^and K^+^ of 31 samples were included in the calculation. Depending on the water quality effect and its importance for human health, the average weight value (AW) between 1 and 5was assigned for each parameter. In the first step, the relative weight (RW) was calculated by using the Eq. (1):

1 $$RW = \frac{{AW}}{{\sum\limits_{{i = 1}}^{n} A W}}$$

The quality rating (Q_i_) was calculated in the second step by dividing the measured parameters (C_i_) to the permitted drinking water values (S_i_) (as per WHO) and multiply by 100 as mentioned in Eq. (2):

2 $${\text{Q}}_{{\text{i}}} = \frac{Ci}{{{\text{Si}}}} X 100$$

In the third step, the Sub-indices (SI) were calculated by using Eq. (3), and WQI was calculated by using Eq. (4).

3 $${SI}_{i}=RW\times {Q}_{i}$$

4 $$WQI=\sum_{i=1}^{n}{SI}_{i}$$

Based on the computed WQI the water was classified into five types: WQI 0–25 excellent, 26–50 good, 51–75 poor, 76–100 very poor, and > 100 unsuitable^27,28^.

## Results and discussion

QGIS SoftwareDesktop 3.18.3 (https://qgis.org/en/site/forusers/download.html) was used to prepare the water quality map in this study based on the selected parameters as shown in Fig. 2a–q. The physicochemical parameters and bacterial analysis results of collected 31 water samples from different locations of Tawangare discussed below. In this study for the reference purpose, permissible limit of different parameters studied were taken from Bureau of Indian Standards (BIS)^29^ and World Health Organization (WHO)^19^.

### Water quality assessment using physio-chemical parameters

The nature of the geological materials through which the surface water flows and the quality of the recharge water defines the types and concentrations of natural contaminants. A wide range of compounds (calcium, magnesium, chloride, arsenate, fluoride, nitrate, iron, etc.) may be picked up by the water when it moves through sedimentary rocks and soils. Hence, the harmful effect of these natural pollutants depends on their type and concentration^12^. The descriptive statistics for the analyzed parameters are summarized in Table 2. The results of the K-S test showed that pH, EC, salinity, Ca^2+^, Mg^2+^and Na^+^ was normally distributed (*p* > 0.05) while Clˉ, Brˉ, NO_2_ˉ, NO_3_ˉ, PO_4_^3^ˉ, SO_4_^2^ˉ, Fˉ, TDS and K^+^ were not normally distributed (*p* < 0.05).

**Table 2:** Statistical summary of the physio-chemical parameters in the study area.

Parameters	Location- Tawang (N = 31)
	Mean ± SD
pH	6.751* ± 0.401
EC (µS/cm)	525.135* ± 32.430
TDS (mg/L)	50.163 ± 33.314
Salinity(mg/L)	50.037 * ± 33.414
Ca (mg/L)	33.13* ± 12.068
Mg (mg/L)	15.53* ± 8.932
K(mg/L)	2.981 ± 2.565
Na (mg/L)	32.55 * ± 11.065
Chloride (Cl^-^) (mg/L)	3.36 ± 4.366
Nitrite (NO_2_^-^) (mg/L)	5.632 ± 6.673
Fluoride (F^-^) (mg/L)	3.980 ± 8.132
Sulfate (SO_4_^-^) (mg/L)	37.104 ± 160.695
Phosphate (PO_4_^-^)(mg/L)	30.447 ± 88.903
Bromide (Br) (mg/L)	3.048 ± 4.7166
Nitrate (NO_3_^-^)(mg/L)	2.692 ± 3.065

*Means are statistically significant (*p* < 0.05).

The acidic or basic nature of any water body is reflected by its pHwhich is one of the most important operational water quality parameters. Since it controls the solubility of various metallic contaminants, it is considered as one of the important parameters of water quality. Discharge of industrial pollutants or human waste in the nearby vicinity, or biological activity are some of the reasonsfor fluctuations in the pH value of any water body. The physico-chemical parameters of water also show a change if the pH of any water body changes due to abovementioned reasons. There are possibilities of the formation of trihalomethanes (toxic) if the pH becomes very high. In the case of alkaline pH, the shifting in pH up over 7 was observed due to the presence of alkaline earth metals that interact with soluble CO forming carbonate and bicarbonates. In the present study, the value of pH from all sources was found to be in the range of 5.96 to 7.43. As per WHO, the permissible limit for pH in drinking water is 8.5^19^, hence the pH of collected water was found to be within this limit.

**Figure 3:**
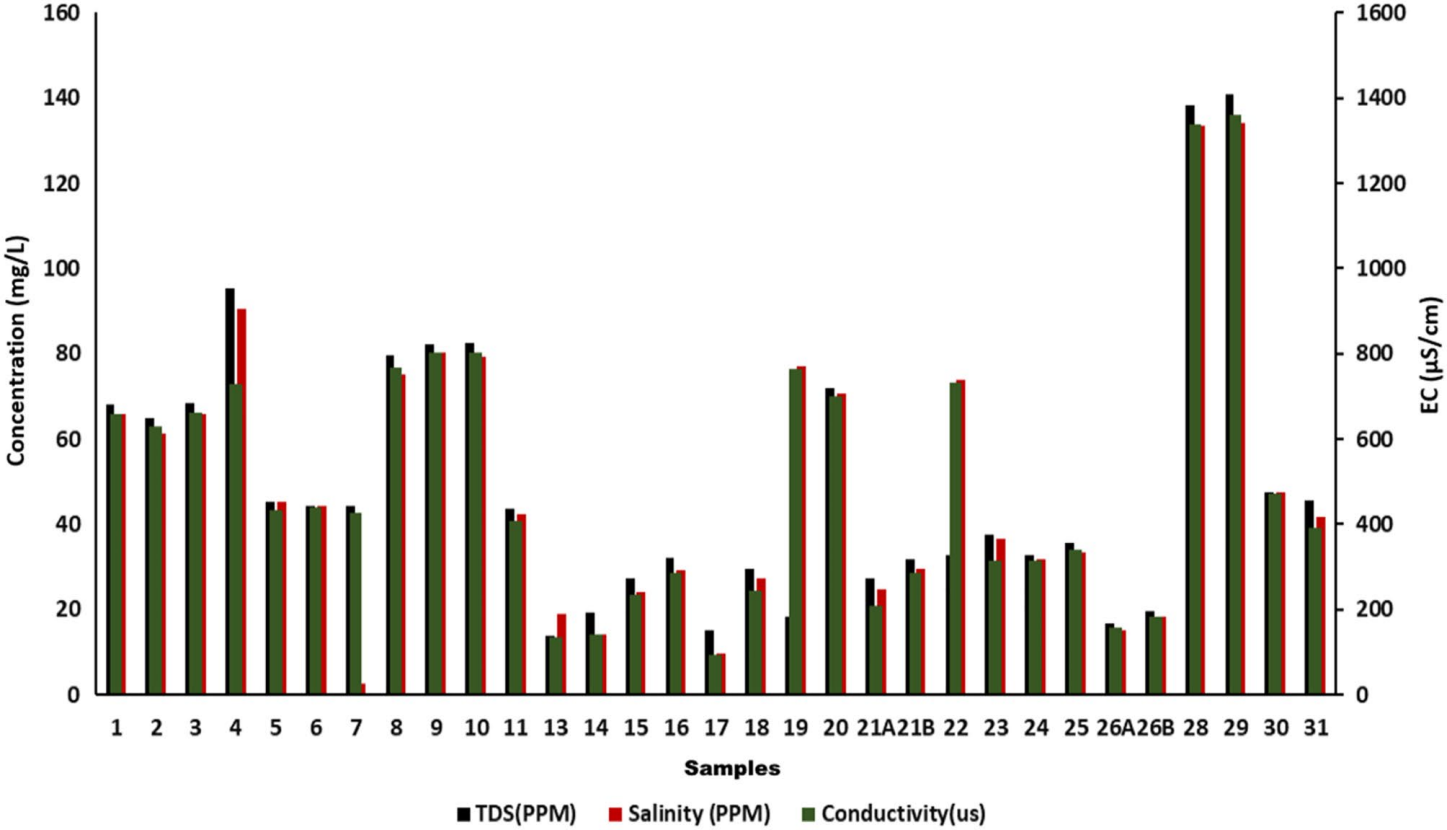
The physio-chemical parameters of 31 water samples collected from Tawang.

The presence of all dissolved solids in water represents TDS (mg/L),along with salinity and conductivity. Additionally, the dissolved organic matter and inorganic salts such as Ca^2+^, Mg^2+^ Na^+^, K^+^, HCO_3_ˉ, Clˉ and SO_4_^2^ˉ in water also contribute to TDS increment**.** Since change in pH affectsthesolubility of suspended matter,itstrongly affects TDS which may sometimes lead to precipitation of some of the dissolved solutes.In the present study, the TDS of the collected water sample varied from 13.85 mg/L to 140.8 mg/L (mean-50.163 mg/L)as represented in Fig. 3. The desirable limit of TDS for drinking water is < 500 mg/L which indicates that the water is in good condition. Tawang water has potable water potential andissuitable for aquatic biota in terms of this parameter. The chief causes of TDS include agricultural operations, domestic runoff, soil contamination caused by leaching, and point source water pollution discharged by industrial or sewage treatment plants^30^.

Electrical conductivity (EC) is the ability of water to conduct electrical current. The increase in the dissolved salt concentration increases the electrical conductivity of water and, therefore, it provides the general indication of water quality with respect to the amount of total dissolved solids in the form of cations and anions, their concentration and mobility, etc. The electrical conductivity varies with temperature as the solubility of salts which areresponsible for ionic composition is also affected by changes in temperature. Hence, in the present study, the electrical conductivity of the collected water samples was found to be in the range of 136.1 µS/cm to 1361 µS/cm as shown in Fig. 3. The average EC value of Tawang samples collected from studied location was 525.13 µS/cm. Though this area is not near to dense urbanization still the EC value is slightly higher thanthepermissible limit (400 µS/cm) this might be due to the natural geological activities^31^.

Since salinity indicates the presence of dissolved salts, it also gives the information of water TDS and conductivity, as these three parameters are interrelated. In addition, it is a key factor limiting biota distribution. If the quantities of dissolved salts in the natural water increase it may lead to severe health issues like high blood pressure (BP) or hypertension leading way to cardiovascular diseases (CVD)^12^. In the present study as shown in Fig. 3, the minimum salinity value was 9.51 mg/L and maximum 134.2 mg/L while the average value was 50.037 mg/L. A statistically significant difference was found between the samples (*p* < 0.05). Because of fewer human activities in this area, the water is found to be within the permissible limit.

Ions such as Clˉ and SO_4_^2^ˉ in water also affect the salinity. The highest Clˉlevel was recordedat site 11 (17.766 mg/L) and the highest SO_4_^2^ˉ was measured 887.54 mg/L for site 1. The results confirm that the anthropogenic activities and hot water springs present in these locations adequatelyimpact the salinity. The Clˉ and SO_4_^2^ˉparameters did not show any significant difference between locations. The average value of Clˉ and SO_4_^2^ˉ in the present study was 3.36 mg/L and 37.104 mg/L respectively. The Clˉion,andSO_4_^2^ˉ ion present in the Tawang region were in permissible limit except for site 1(above permissible limit). In practice salinity is mostly the result of the following major inorganic ions such as Na^+^, Ca^2+^, Mg^2+^, K^+^, Clˉ, SO_4_^2^ˉ_,_ etc. Water quality is also decreased by increasing the nitrogenous and phosphorous compounds in it. The increase in these compounds might be due to the excessive use of fertilizers in farming which are mixed with the river stream by runoff due to rainy climate.This event is known as primary contamination which may lead to eutrophication giving rise to secondary contamination, threatening the biodiversity in water^12^. The highest NO_3_ˉ was recorded to be 32.15 mg/L for site 9 while the highest NO_2_ˉ was measured at sight 6 (8.86 mg/L). These two ions in 31 sites of Tawangwere in the permissible limit (50 mg/L)as per WHO. However, the PO_4_^3^ˉvalues in sites 2, 26B, and 30 were 378.9,88.39 and 322.88 respectively which is above the permissible limit (40 mg/L). The fluoride ions in the Tawang region in some sites (3, 16, 23, 24, 26A, and 28) were above the permissible limit (1.5 mg/L). This might be due to fluoride-bearing minerals present in rocks and sediments. In addition, the use of pesticides in agriculture might be increasing the fluoride concentration in these sites. Table 3 shows the values of ions present in 31samples from different locations which were identified by ion chromatography.

**Table 3:** List of anions detected by ion chromatography.

Sample	Chloride	Nitrite	Fluoride	Nitrate	Sulfate	Phosphate	Bromide
1	0	0	0	0	887.544	0	0
2	0	5.692	0	0	10.307	378.96	11.62
3	7.554	5.22	20.892	5.059	10.385	0	12.348
4	0	0	0	3.542	4.926	11.64	12.563
5	6.21	3.88	0	4.22	9.83	13.31	0
6	7.285	8.861	0	0	2.71	12.206	0
7	2.491	5.253	0	12.581	5.859	12.177	4.22
8	0	0	0	0	10.367	0	0
9	0.436	0	0.036	32.159	7.458	2.92	0
10	7.725	0	0	9.876	4.985	12.177	6.914
11	17.766	0	0	4.865	10.16	0	0
13	0.788	0	0	0	11.138	0.099	0
14	0	0	0	3.476	15.364	1.998	0
15	3.961	7.816	0	12.28	0	8.879	2.834
16	0	5.291	19.256	0	9.559	13.151	0
17	0.326	0	0	3.071	23.219	1.625	0
18	0.34	0	0.307	6.009	5.207	0.893	0
19	4.899	7.55	0	10.877	6.375	10.161	0
20	0.925	0	0	2.187	3.799	1.001	0
21A	6.208	0	0	11.025	0.882	8.934	2.868
21B	0	4.946	0	14.824	6.93	11.532	0.519
22	0	0	0.26	0	6.355	0.242	0
23	8.777	5.539	21.12	4.69	10.173	0	0
24	8.085	5.488	21.863	5.049	10.283	0	11.715
25	0.425	0	0	4.146	10.625	0.241	0
26A	0	4.722	14.288	4.381	10.135	0	3.704
26B	0	0	0	9.514	11.312	88.39	0
28	7.777	4.963	21.395	0	0	0	11.022
29	0	0	0	0	7.262	0	0
30	8.839	5.563	0	5.152	0	322.887	11.141
31	2.7.4	8.089	0	14.6	0.158	11.787	1.471

Turbidity is the easiest measure of water quality for any human as it shows how clean or cloudy water is. Several factors affect the turbidity of any water body, and it is caused by particles (clay, silt, phytoplanktons algae, fine organic and organic matter, inorganic matter, and other microscopic organisms, etc.) which are suspended or dissolved in water that scatter light making it appear cloudy. The presence of many suspended solids indicates the high turbidity which reduces the aesthetic quality of any water source^32^. Additionally, turbidity also increases the cost of the water treatment process from different industries such as food processing, pharmaceutical, etc. The main sources of turbidity are natural (erosion from uplands, stream channel movement, etc.) or anthropogenic activities (rock blasting or digging also cause erosion). Turbidity may not be intrinsically harmful, but it interferes with disinfection during water treatment and provides a medium for microbial growth. This leads to serious consequences such as nausea, cramps, diarrhea, etc.^8,9,21^. In the present study, the water samples collected from Tawang, India showedaturbidity value of 0. This might be due to weather conditions as the samples were collected during the winter season. During this season the average rainfall in the Tawang area is 53.3 mm which is very little as compared to average rainfall during the rainy season (1723 mm). Moreover, in this season the soil leaching is very much negligible and mostly water sources in frozen condition.

**Figure 4:**
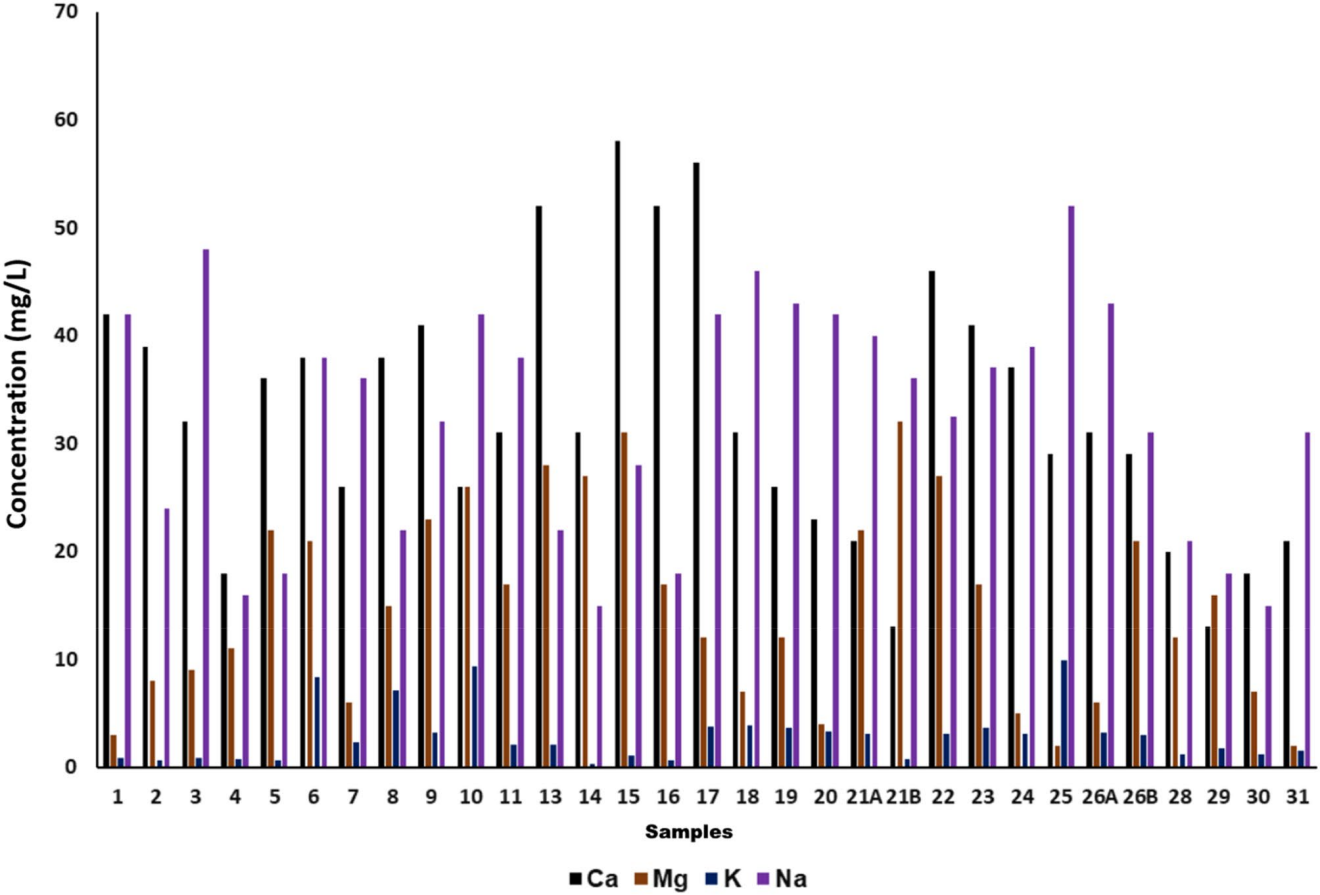
Calcium (Ca^2+^), Magnesium (Mg^2+^), Sodium (Na^+^) and Potassium (K^+^) of 31 water samples collected from Tawang.

The dissolved polyvalent metallic ions from sedimentary rocks, seepage, and runoff from soils are the principal natural sources of hardness in water. Hard water is very dangerous to human health as it causes many diseases such as osteoporosis, nephrolithiasis, hypertension, stroke, etc. It occurs mainly due to the salts of magnesium and calcium. Though these are the common essential mineral constituents of food,their excess intake can cause various abovementioned diseases. Hardness in water is expressed in terms of milligrams of calcium carbonates equivalent per liter^12,33^. Water containing calcium carbonate below 60 mg/L is considered as soft water while containing 60–120 mg/Lis moderately hard and having 120–180 mg/Lis hard, and more than 180 mg/Lis considered to bevery hard water. Therefore, in the present study, the calcium was in the range between 13 mg/L and 58 mg/L and magnesium was in the range between 2 mg/L and 32 mg/L as mentioned in Fig. 4. According to these results, the water of the Tawang area is soft as the values comply with national and international drinking water standards such as BIS-IS-2500 (2012), WHO, and EU^18,19,29^. The mean values of Ca^2+^ (33.13 ± 12.06 mg/L) and Mg^2+^ (15.53 ± 8.93 mg/L) in the study area are within the permissible limit.

One of the most abundant elements of the earth’s crust is iron. Natural or anthropogenic sources can be the cause of iron contamination in water. The iron contaminated water not only leads to staining of laundry and utensils but also reacts with tea and coffee producing black color. Iron is an essential element and structural component of hemoglobin, myoglobin,several enzymesetc, and its deficiency leads to anemia and in severe cases loss of well-being. In addition, its overdose in humans can cause severe health problems such as liver cancer, diabetes, cirrhosis of the liver, heart disease, infertility, etc. The high concentration of iron in water changes its color, taste, odor, and also corrodes water pipelines^12,33^. In the present study ironcontamination of the collected water samples was found to be in the range of the permissible limit.

### Principle component analysis

Principle component analysis (PCA) is used to reduce the dimensions of multivariate datasets. While trying to reduce the input data dimensions, PCA retains the maximum informative value of the input data sets. PCA decreases the number of dimensions that are not correlated and summarizes the information that is dispersed in several dimensions. PCA discards the redundant and highly correlated parameters and selects the independent variables. Additionally, it also identifies the variance in terms of a small number of new pseudo-variable (Principle component) within a huge dataset of correlated variables^9,34^.

Kaiser–Meyer–Olkin (KMO) and Bartlett’s tests of Sphericity have been performed to examine the suitability of the present dataset for PCA. Sampling adequacy is measured by KMO which indicates that the proportion of variance is caused by underlying Principal Components^22,35^. A value closer to 1 generally indicates that the data sets may be used for PCA and in this study KMO value is 0.547. If the KMO value < 0.5 then the data sets will not be useful for PCA. To examine whether the correlation matrix is an identity matrix, Bartlett’s test of Sphericity was used. All variables become unrelated making the PCA model inappropriate and unsuitable statistical tool for advanced data analysis if the correlation matrix is an identity matrix. The Null Hypothesis of Bartlett’s test assumes that there is no scope for dimensionality reduction (if the correlation matrix is the identity matrix). In the present work, the significance level (0.000) is less than 0.05 which rejects the Null Hypothesis. Therefore, it means that there is no significant relationship among the parameters. Finally, using Varimax rotation with Kaiser Normalization PCA has been carried out on normalized data and so the covariance matrix coincides with the correlation matrix (Table 4). SPSS software was used to carry out both tests. The % variance and cumulative variance is also represented in Table 4. This explains more than 76% of the total variance and shows only the first six components. However, more components might be needed as the first component by itself explains less than 25% of the variance. In the standardized ratings, six PCs explain more than 76% of the total variability so these PCs have been reasonably retained to reduce the dimension further.

**Table 4:** Principle component analysis. Rotated component matrix (Varimax rotation).

	Components					
	PC1	PC2	PC3	PC4	PC5	PC6
Cl^−^	.085	.622	.025	.233	.007	.242
NO_3_^−^	−.195	.684	.219	−.162	−.086	.056
F	.084	.802	−.198	−.111	.246	−.175
NO_2_^−^	−.150	.066	−.095	−.085	−.683	.232
SO_4_	.045	−.228	−.194	−.145	.107	−.776
PO_4_^−^	−.051	−.050	.883	−.212	.101	.052
Br	.308	.516	.585	−.111	.078	−.064
pH	−.023	.276	.169	.181	.706	.084
EC	.962	.014	.013	−.014	−.006	−.032
TDS	.954	.020	.060	−.071	.001	−.060
Salinity	.964	−.001	−.008	−.041	.038	−.005
Ca	−.467	−.048	−.355	−.142	.609	.004
Mg	−.105	−.202	−.419	−.304	−.055	.708
K	.034	−.181	−.104	.864	.097	.176
Na	−.254	.160	−.220	.751	−.161	−.367
Eigen Value	3.435	2.275	1.775	1.545	1.323	1.084
Variance (%)	22.899	15.165	11.830	10.30	8.823	7.224
Cumulative (%)	22.899	38.064	49.894	60.018	69.018	76.242

Extraction method: Principal Component Analysis.

Rotation Method: Varimax with Kaiser Normalization.

**Figure 5:**
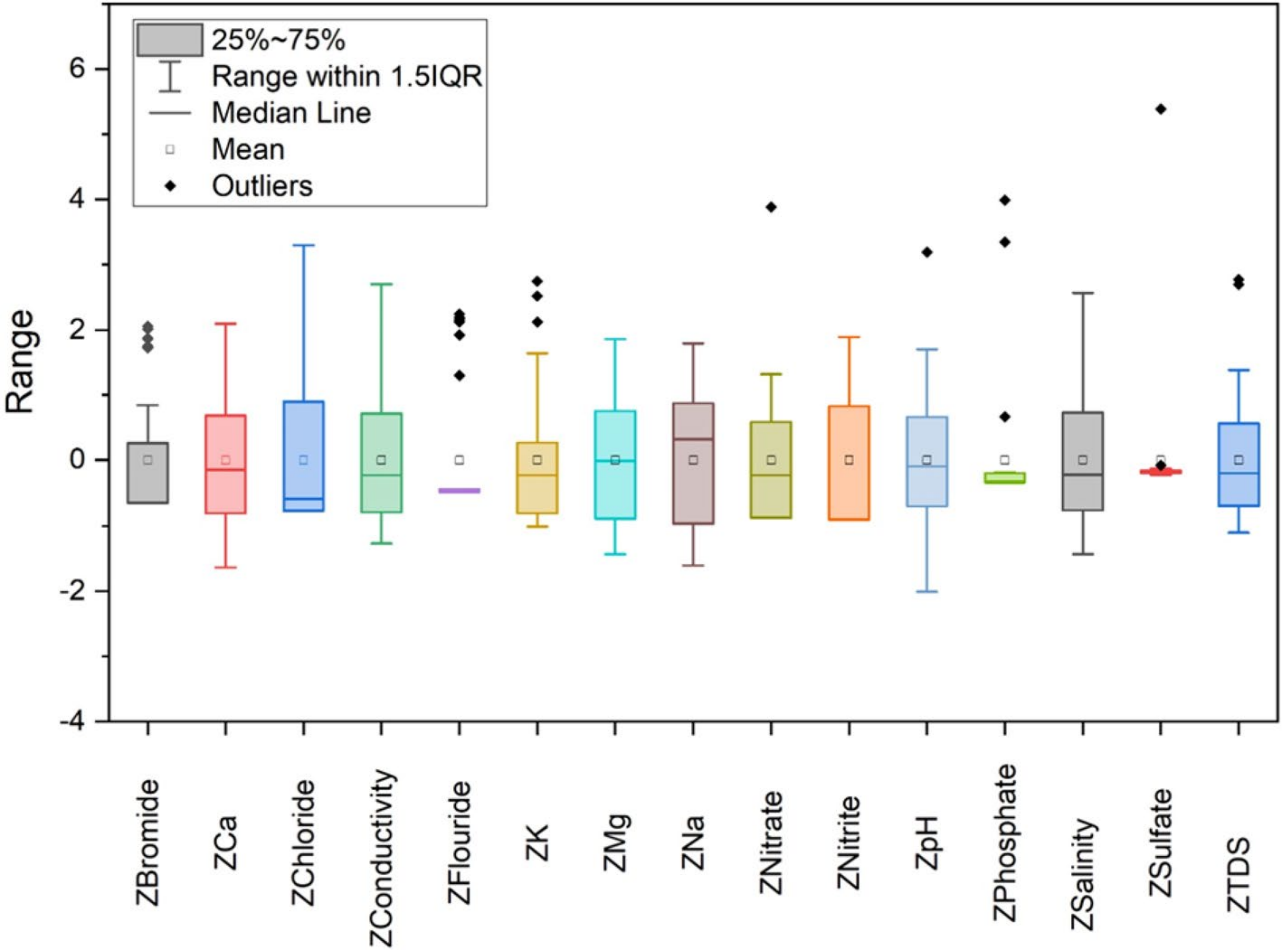
Box plot shows the z-score variability of data.

The z-score analysis has been performed for 15 parameters in this study. Figure 5 shows the Box and whisker plot which displays the variability in z-score values of the data points. The maximum and minimum values in the box plot are represented via vertical black lines which in most cases lie beyond the boxes. The whiskers that are not considered outliers are extended to the most extreme data points and the outliers in the box plot are plotted individually using the ‘ + ’ symbol. To obtain a set of linearly transformed scores, Z-scores are estimated which can be simplified by plotting a straight-line graph between z-scores and corresponding scores. The parameters were subjected to PCA after the estimation of z-scores. Analysis of 15 parameters would still be a very tedious and expensive job so PCA has been performed which reduced the parameters using a statistical approach. PCs are independent axes where data is projected^22,36^. Table 3 shows the rotated factors loading, eigenvalues, individual variance, and cumulative variance of these PCs. The 6 PCs of these parameters account for 76.24% of the total variance and have individual eigenvalues > 1.

**Figure 6:**
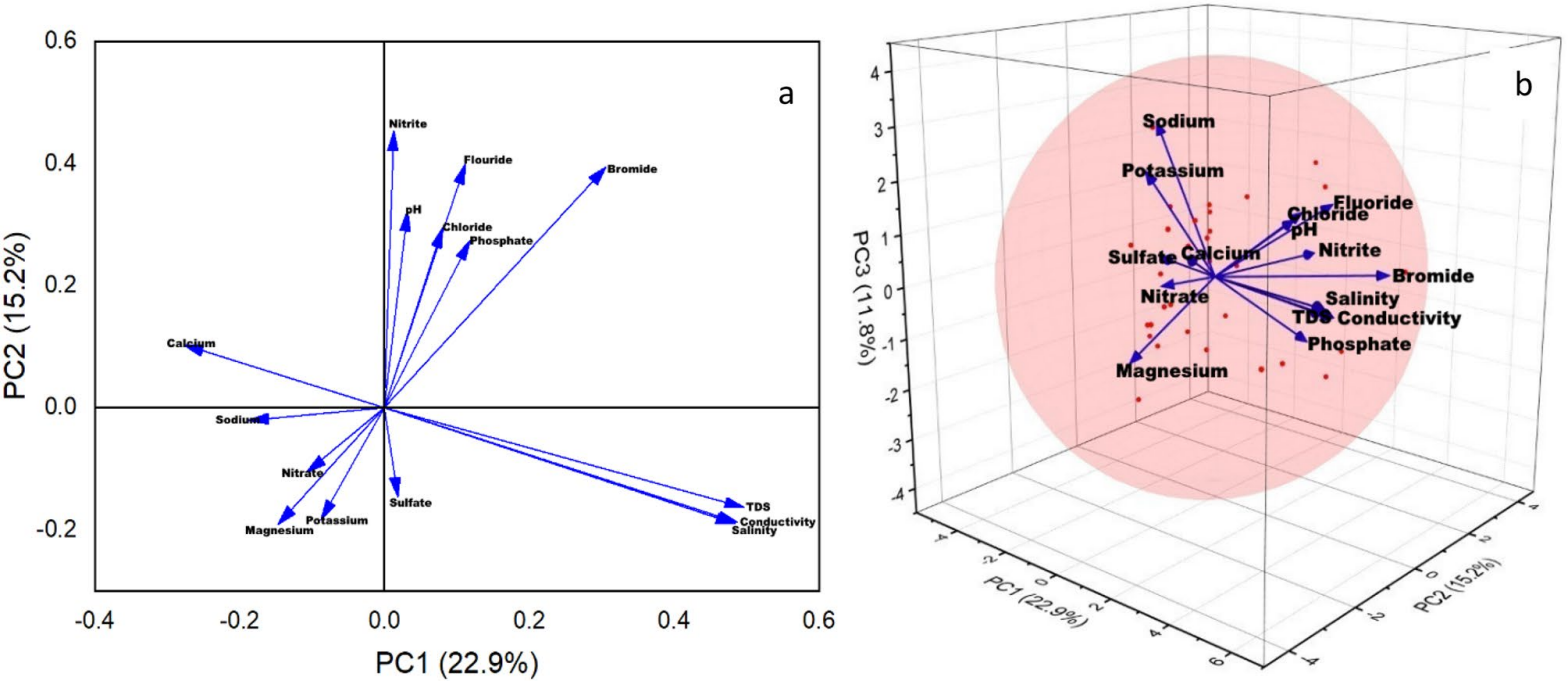
(**a**) 2D biplot (2PCs) and (**b**) 3D plot (3PCs) of principal components.

Figure 6 shows the relation between the first 2 and 3PCs that contributes maximum to the overall variance. The direction and length of the vectors in the 2D bi-plotare represented in Fig. 6a which indicates the contribution of each variable to the two PCs in the bi-plot. For example, on the horizontal axis, the first PCs have positive coefficients for Chloride, Fluoride, Sulphate, Bromide, EC, TDS, Salinity, and Potassium while negative coefficients for nitrate, nitrite, phosphate, pH, calcium, magnesium, and sodium. That is why 8 vectors are directed into the right half of the plot and 7 are directed to the left half of the plot. Similarly, the PC2 has negative coefficients on the vertical axis for sulfate, phosphate, salinity, calcium, magnesium, and potassium whereas has positive coefficients for the remaining 9 variables. A point for the mean values of each parameter at all of the 31 locations was also included in this 2D biplot. Therefore, their relative locations can be determined from the plot as these points are scaled with respect to the maximum score value and maximum coefficient length. If the first 2 PCs do not explain enough of the variance in the data, then the 3-dimension plot proves to be quite useful. Figure 6b shows the 3D representation of first 3 PCs.

### Correlation analysis

Pearson linear correlation matrix was generated by using those parameters which were contributingamaximum of 6 PCs (> 0.35). Table 5 shows the most effective water quality parameters to define any co-variation. The obtained resultsindicateavery strong positive correlation between TDS and EC, and salinity. Salinity shows a strong positive correlation with TDS. pH shows weak and very weak positive correlation with all parameters except NO_2_ˉ. While Ca has a very weak positive correlation with NO_3_ˉ, Fˉ and pH; it has a very weak negative correlation with Clˉ, NO_2_ˉ, PO_4_^3^ˉ and Br and has a moderate negative correlation with EC, TDS, and salinity. A similar very weak negative correlation is shown between K^+^ and all the parameters except Clˉ, pH, and EC (shows very weak positive correlation). Na shows a very weak negative correlation with NO_3_ˉ, PO_4_^3^ˉ, Brˉ, EC, TDS, and salinity; it has a very weak positive correlation with Fˉ, Clˉ, NO_2_ˉ and pH.Brˉ and moderate positive correlation with Fˉ and PO_4_^3^ˉ and shows very weak negative correlation with NO_2_ˉ. Hence, correlation coefficients between pairs of water quality parameters concentrations indicate that Fluoride, Bromide, phosphate, and calcium values significantly correlate with pollutant parameters such as nutrient and trace elements. This suggested that these nutrients and trace elements have high values as they are significantly affecting TDS, salinity, and EC because ofthenatural geological activities in the study area.

**Table 5:** Pearson correlation matrix among the variables.

	Cl^-^	NO_3_^-^	F	NO_2_^-^	PO_4_^-^	Br	pH	EC	TDS	Salinity	Ca	K	Na
Cl^-^	1												
NO_3_^-^	.282	1											
F	.278	.366*	1										
NO_2_^-^	.004	.086	−.201	1									
PO_4_^-^	.021	.228	−.157	−.103	1								
Br	.265	.275	.398*	−.099	.458**	1							
pH	.184	.131	.231	−.179	.114	.226	1						
EC	.051	−.075	.059	−.095	.009	.267	−.009	1					
TDS	.044	−.147	.101	−.102	.018	.360*	−.002	.907**	1				
Salinity	.055	−.118	.071	−.134	.002	.270	.006	.968**	.889**	1			
Ca^2+^	−.124	.024	.113	−.137	−.093	−.266	.292	−.384*	−.417*	−.363*	1		
K^+^	.063	−.171	−.163	−.003	−.211	−.224	.032	.015	−.037	−.001	.023	1	
Na^+^	.134	−.027	.072	.193	−.322	−.153	.023	−.210	−.297	−.241	.023	.496**	1

Signifance values are in bold.

*Correlation is significant at the 0.05 level (2-tailed).

**Correlation is significant at the 0.01 level (2-tailed).

### Biological parameters for water quality assessment

A wide variety of pathogenic and non-pathogenic microorganisms are found in water bodies which may lead to unpleasant taste and odour in water. This may be served as an indicator and the main concern behind studying the microbiological quality of water. Bacteria, helminths, protozoa, and viruses are contaminants that are derived from feces, household waste, etc. Indicator organisms are generally used to analyze the microbiological quality of water and among them, coliform and *E. coli* are such indicators. In this work, the bacterial colony count of collected water samples from different locations of Tawang was found to be in the range of 7 CFU/ml to 20117 CFU/ml as mentioned in Table 7. As per BIS Standards^29^ the permissible limit of bacterial count in drinking water should be 0 CFU/100 ml.

### Spatial distribution pattern

Figure 2a–g represents the spatial distribution pattern of different water quality parameters in the location maps of sample collection area. Figure 2a represents the spatial distribution pattern of the pH, which indicates that the central part along with NE-WS across the district shows the alkaline nature of surface water. On the other hand the NE-NW along with the central part represents the acidic nature of water. According to previous reports, fluoride is absorbed on a clay surface in acidic water,and desorbed in alkaline water. Hence, for fluoride dissolution, alkaline pH is more favorable^37,38^. The EC in the district is in the range of 136.1µS/cmto 1361µS/cm(Fig. 2b). The TDS is also low in the central and NE-NW-SE-SW parts of the Tawang (Fig. 2c). This shows that the EC and TDS have a significant positive correlation as evidence by the correlation matrix of the quality parameters (Table 5). The salinity map clearly and significantly indicates that it is within the permissible limit (600 mg/L) but unevenly distributed in the Tawang district (Fig. 2d). A positive correlation is observed between the salinity, EC, and TDS of the surface water as mentioned in Table 5. Most of the surface water in the spatial distribution graph showed alkaline nature which might be due to the presence of carbonatesand bicarbonates^39^.

The spatial distribution of Ca^2+^ and Mg^2+^ suggested varying concentrations within the permissible limit which reflects that the study area is characterized by soft surface water (Fig. 2g and h). The study area is surrounded by rocks covered with snow, so the Ca^2+^ and Mg^2+^ ions present in the surface water might be due to the leaching of calcium and magnesium-bearing rocks. Ca^2+^shows a significant negative correlation with EC, TDS, and salinity. Na^+^is highest (within permissible limit) in the central part and some patches in the NW part of the district. It is also showing negative correlation with the TDS and salinity in the spatial distribution pattern and correlation matrix. The presence of K^+^ is within thepermissible limit but it is covering the major portion of the district (Fig. 2i)^40^. The spatial distribution pattern of sulphate (Fig. 2q) and chloride (2 k) reveals that they are present within the permissible limit. The spatial distribution pattern of iron reveals its absence in the district (Fig. 2j). Another important factor for the study area is fluoride (Fig. 2m) which is mainly observed in the SE part of the district above the permissible limit, with a concentration of more than 10 mg/L which may lead to skeletal fluorosis^41^. Fluoride concentration in surface water depends on many factors such as temperature, pH, the solubility of fluorine bearing minerals, size, and type of geological formations, presence, and absence of complexing or precipitating ions and colloids, anion exchange capacity of water and the contact time during which the water remains in contact with the geological formations^12,40,42^.

Nitrate, nitrite, and phosphate in the surface water are mainly due to anthropogenic activities such as waste disposal, sanitary landfills, overapplication of fertilizers or improper manure management practices,etc.^43^. In this study, it was observed that the nitrate, nitrite, and phosphate are within the permissible limit which indicates absence of any such kind of activities (Fig. 2n,o,p).

### Water quality assessment using WQI

FOR A RAPID ASSESSMENT OF ENVIRONMENTAl impact,WQI can help us to decide overall water quality. WQI provides a value with a quick and understandable explanation of water quality. BIS^29^, US EPA^44^,and WHO standard^19^ parameter values were used for the calculation of WQI at different water sampling locations in Tawang, Arunachal Pradesh. The relative water quality parameters are presented in Table 6.

**Table 6:** Relative weight (RW) of each parameter.

Parameters	Water quality standards	Assigned weight (AW)	Relative weight (RW)
Chloride (Cl^-^) (mg/L)	250*	2	0.055
Nitrite (NO_3_^-^)(mg/L)	50*	2	0.055
Fluoride(mg/L)	1.5*	3	0.083
Nitrate (NO_2_^-^)(mg/L)	50*	2	0.055
Sulfate (SO_4_) (mg/L)	500*	1	0.027
Phosphate(mg/L)	40***	3	0.083
Bromide (Br) (mg/L)	1***	3	0.083
pH	8.5**	4	0.111
Electrical Conductivity (EC) (µS/cm)	400*	3	0.083
TDS (mg/L)	500**	3	0.083
Salinity(mg/L)	600***	3	0.083
Calcium (Ca) (mg/L)	75**	3	0.083
Magnesium (Mg) (mg/L)	30**	2	0.055
Potassium (K) (mg/L)	8***	1	0.027
Sodium (Na) (mg/L)	200**	1	0.027

*WHO (2017), **BIS (1991), ***US EPA (1998).

In the present study, the water samples were collected during the winter season, and the calculated WQI results are mentioned in Table 7. The average WQI value in Tawang is 82.49. The WQI results of maximum samples are in the range of 0–50 which are considered as good for drinking, while some samples are unsuitable for drinking showing value > 50. In terms of WQI, the Tawang water samples from most of the sites have good water quality and some have poor water quality. Therefore, WQI has been widely applied in the monitoring of water quality and plays a significant role in water resource management for suitable applications.

**Table 7:** WQI, bacterial count (CFU/ml) and water type of Tawang samples collected from different sites.

Site	WQI	Bacterial count (CFU/ml)	Status	Water Type	Site	WQI	Bacterial count (CFU/ml)	Status	Water Type
1	32.69	30	Good	Na^+^–Cl^-^	18	22.05	7	Excellent	Na^+^–HCO_3_^-^
2	204.16	2083	Unsuitable	Ca^2+^–Mg^2+^–HCO_3_^-^	19	33.86	22	Good	Na^+^–HCO_3_^-^
3	248.49	1285	Unsuitable	Na^+^–HCO_3_^-^	20	27.76	25	Good	Na^+^–HCO_3_^-^
4	135.90	2018	Unsuitable	Ca^2+^–Mg^2+^–HCO_3_^-^	21A	47.86	29	Good	Na^+^-HCO_3_^-^
5	29.72	37	Good	Ca^2+^–Mg^2+^–HCO_3_^-^	21B	31.17	35	Good	Ca^2+^–Mg^2+^-HCO_3_^-^
6	33.01	40	Good	Ca^2+^–Mg^2+^–HCO_3_^-^	22	36.16	42	Good	Ca^2+^–Mg^2+^-HCO_3_^-^
7	61.62	511	Poor	Na^+^-HCO_3_^-^	23	143.83	11,653	Unsuitable	Ca^2+^–Mg^2+^-HCO_3_^-^
8	33.04	33	Good	Ca^2+^–Mg^2+^–HCO_3_^-^	24	243.13	13,975	Unsuitable	Na^+^–HCO_3_^-^
9	39.44	46	Good	Ca^2+^–Mg^2+^–HCO_3_^-^	25	24.38	11	Excellent	Na^+^–HCO_3_^-^
10	97.28	610	Very Poor	Na^+^–HCO_3_^-^	26A	129.20	20,117	Unsuitable	Na^+^–HCO_3_^-^
11	25.82	10	Excellent	Na^+^–HCO_3_^-^	26B	40.37	44	Good	Ca^2+^–Mg^2+^-HCO_3_^-^
13	24.02	14	Excellent	Ca^2+^–Mg^2+^–HCO_3_^-^	28	252.23	10,642	Unsuitable	Ca^2+^–Mg^2+^-HCO_3_^-^
14	20.62	10	Excellent	Ca^2+^–Mg^2+^–HCO_3_^-^	29	41.26	28	Good	Ca^2+^–Mg^2+^-HCO_3_^-^
15	54.14	340	Poor	Ca^2+^–Mg^2+^–HCO_3_^-^	30	184.07	11,685	Unsuitable	Ca^2+^–Mg^2+^-HCO_3_^-^
16	134.08	1021	Unsuitable	Ca^2+^–Mg^2+^–HCO_3_^-^	31	37.85	20	Good	Na^+^–HCO_3_^-^
17	23.57	11	Excellent	Ca^2+^–Mg^2+^–HCO_3_^-^					

In this research, the WQI value calculated for sites 13, 14, 17, 18, and 25 was 24.58, 21.22, 23.96, 23.07 and 25.80 respectively. These results show that the water of these sites is suitable for drinking as it comes in the range of 0–25. Additionally, the WQI value calculated for site 1, 5, 6, 8, 9,11, 19, 20, 21A, 21B, 22, 26B, 29, and 31 was in the range of 26–50 which shows that the water from these sites is suitable for drinking after normal treatment. However, the WQI values in sites 2, 3, 4, 10, 16, 23, 24, 26A, 28, and 30 are more than 100. Because of the water flowfrom agricultural land and household wastewater into these sites,adecrease in water quality is observed leading to high WQI values.

### Hydrogeochemical facies and rock water interaction

**Figure 7:**
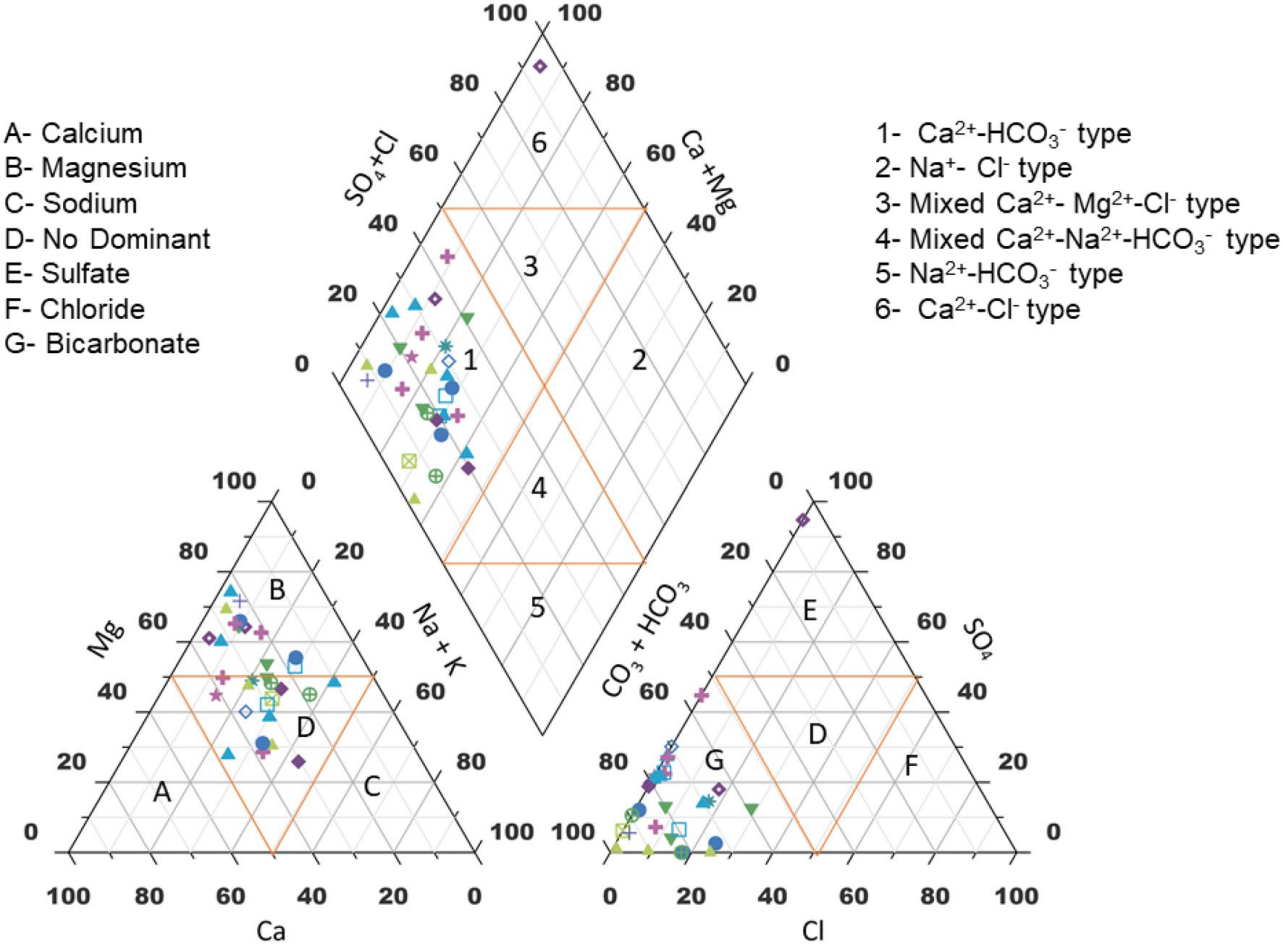
Hill Piper trilinear diagram.

Based on their hydrogeochemical facies, Hill Piper trilinear diagram explains and classifies different types of water groups^45^. Figure 7 shows the uneven distribution of major ions which are plotted on the Hill-Piper trilinear diagram using Grapher Software (Grapher 16.3.410)( graphersupport@goldensoftware.com). This diagram represents the major significant cations and anions responsible for the nature of surface water. It is comprised of two triangles at the base and one diamond shape at the top which categorized surface water into various six types (Ca^2+^- HCO_3_ˉ type, Na^+^- Clˉ type, Mixed Ca^2+^- Mg^2+^- Clˉtype, Mixed Ca^2+^-Na^+^- HCO_3_ˉ type, Na^+^- HCO_3_ˉ type and Ca^2+^- Clˉtype)^46^. A critical evaluation of the diagram reflects that majority of the samples (50%)fall under Ca^2+^- HCO_3_ˉtype and 10% of the samples showed Ca^2+^-Clˉ type. The study was conducted during winter season and from the results it is clear that weathering of rocks and precipitation are the major processes occurring in the surface water environment. Hydrochemistry of the investigated samples represents that the alkaline earth > alkali metals and weak acid > strong acidic anions. Major cations are present in order, No Dominant type ˃ Mg^2+^of the mean abundance while anions are present in mean abundance order of HCO_3_ˉ˃SO_4_^2^ˉ. Hence, it can be concluded that the surface water in this study is polluted due to natural activities or rock-water interaction. Similar type of studies have been carried out to find the hydrogeochemistry and water quality ofRewalsar Lake of Lesser Himalayan,which showed that the alkaline earth surpass the alkaline metal and weak acid exceed to strong acid ^5,47^.

**Figure 8:**
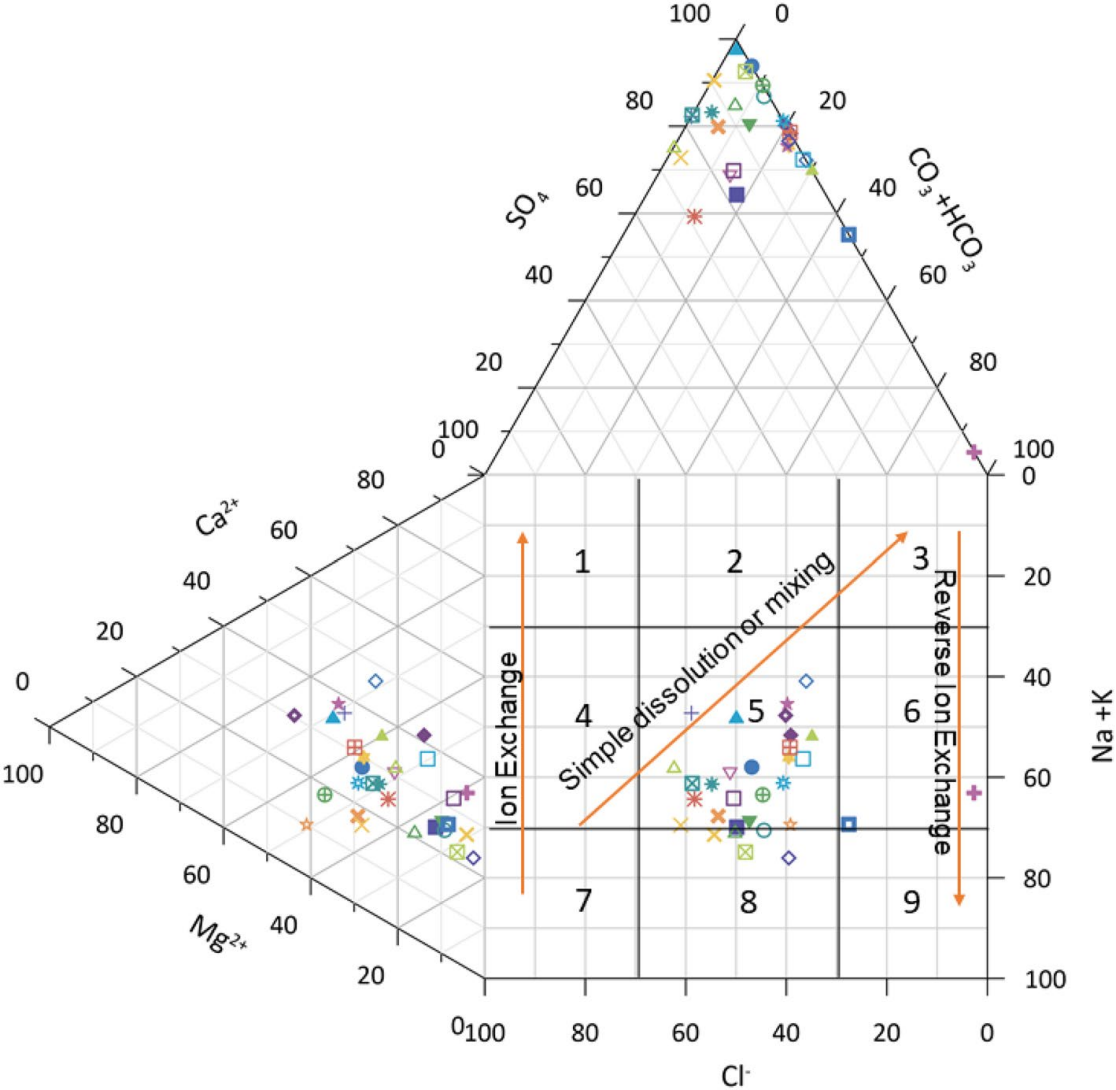
Durov plot illustrating hydrochemical processes involved in surface water/groundwater in different locations of Tawang area.

The geochemical processes occurring in the surface water of the study area was further verified by Durov’s plot (Grapher16.3.410, graphersupport@goldensoftware.com)^48^. This diagramconsistsof two ternary plotswhich are plotted against anions and cations of interest (data are normalized to 100%). The Durov plot reveals the relationship and properties of large sample groups while clustering the data points indicating the samples with similar chemical composition^49^. Figure 8 shows the Durov’s plot which evaluate the water type from geochemical process that affect the surface water. In this study, most of the surface water samples are plotted in field 5 which indicates that water exhibits simple dissolution or mixing (no dominant of cation or anion). Some of the samples fall in field 8 which indicates that the surface water has undergone reverse ion exchange with water minerals. Few of the surface water samples are plotted in field 6 which indicates the probable mixing or uncommon dissolution influences (SO_4_^2-^ dominant or anion discriminant and Na dominant). In recent research on Parbati river overall water quality, hydrogeochemical characteristics and other chemical parameters were assessed. The results of Durov’s plot of this study are in line with the present research^50^.

Hydrogeochemistry of water is altered and affected by different natural processes like evaporation, precipitation, rock weathering and combination of all these processes. These processes can be deciphered using Gibb’s diagram which is characterized by three chief zones: rock weathering dominance, precipitation, and evaporation. It is formed by plotting between the ratio of TDS vs Clˉ/(Clˉ + HCO_3_ˉ) and TDS vs Na^+^/ (Na^+^  + Ca^2+^) for anions and cations respectively whereby all ions are expressed in meq/L.It is well known that the reactions occurinsurface water and essential minerals of the water play a vital role towards water quality. Additionally, it is also useful in knowing the primary mechanism of ion contribution in surface water ^46,51^.

**Figure 9:**
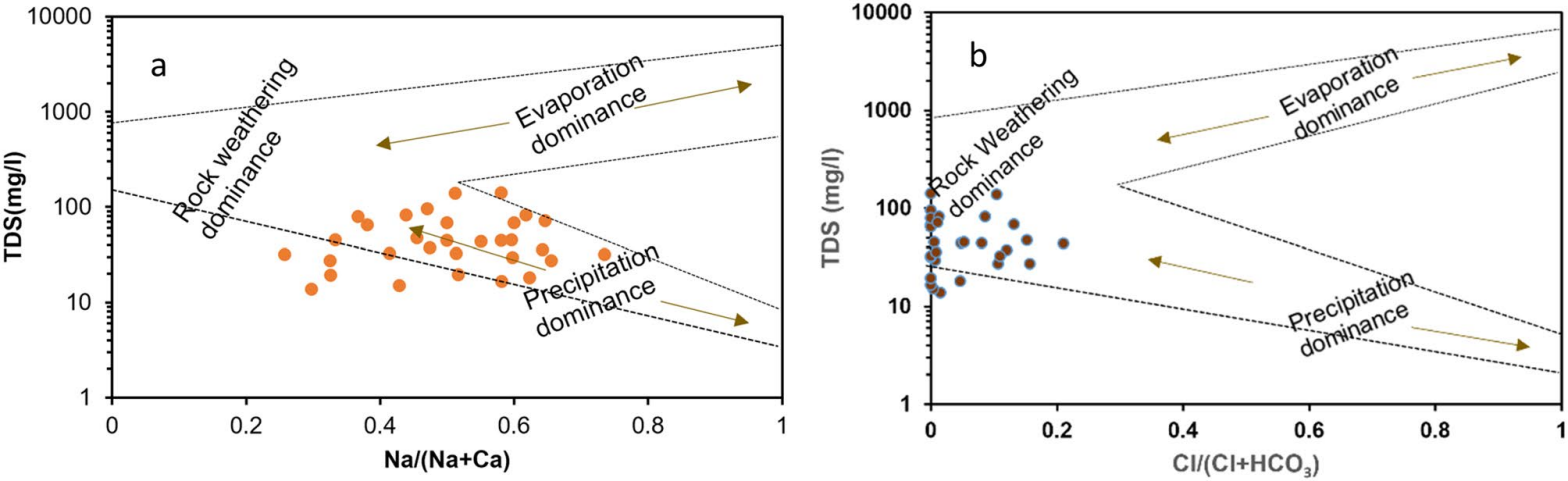
Gibbs diagrams (**a**) cations: TDS vs Na^+^/Na^+^  + Ca^2+^) (**b**) anions: TDS vs Cl/(Cl^-^ + HCO_3_^-^)reveals water chemistry controlling mechanism.

The Gibbs plot of surface water samples collected from Tawang area in winter season has been shown in Fig. 9a and b. The samples collected from this area is prevalent with rock-water interaction and precipitation. Therefore, in plot 10a precipitation dominates over rock -water and evaporation while in plot 10b, rock- water interaction dominates over evaporation and precipitation. Hence, in this study the two primary factors that influenced the surface water chemistry are weathering of rock forming minerals and rainwater intrusion into the aquifer. The samples which lie in the evaporation zone are the indicative of the water influenced by sea water and in this study the samples have fallen into precipitation or rock- water dominant zone. While if any sample falls outside these three mentioned zones, then might be due to any anthropogenic activities.

Earlier studies on different water bodies in Himalayan regions also confirmed rock dominance as a main factor for controlling ionic composition^47,52,53^. The Himalayan water bodies are surrounded by rocks so the water percolation through the rocky lithology and longtime rock water interaction may result in high solute concentration. Furthermore, the less ionic composition in this study reflects the precipitation dominance which might be due to precipitation and melting of ice at higher altitude of Himalayan region^52^.

**Figure 10:**
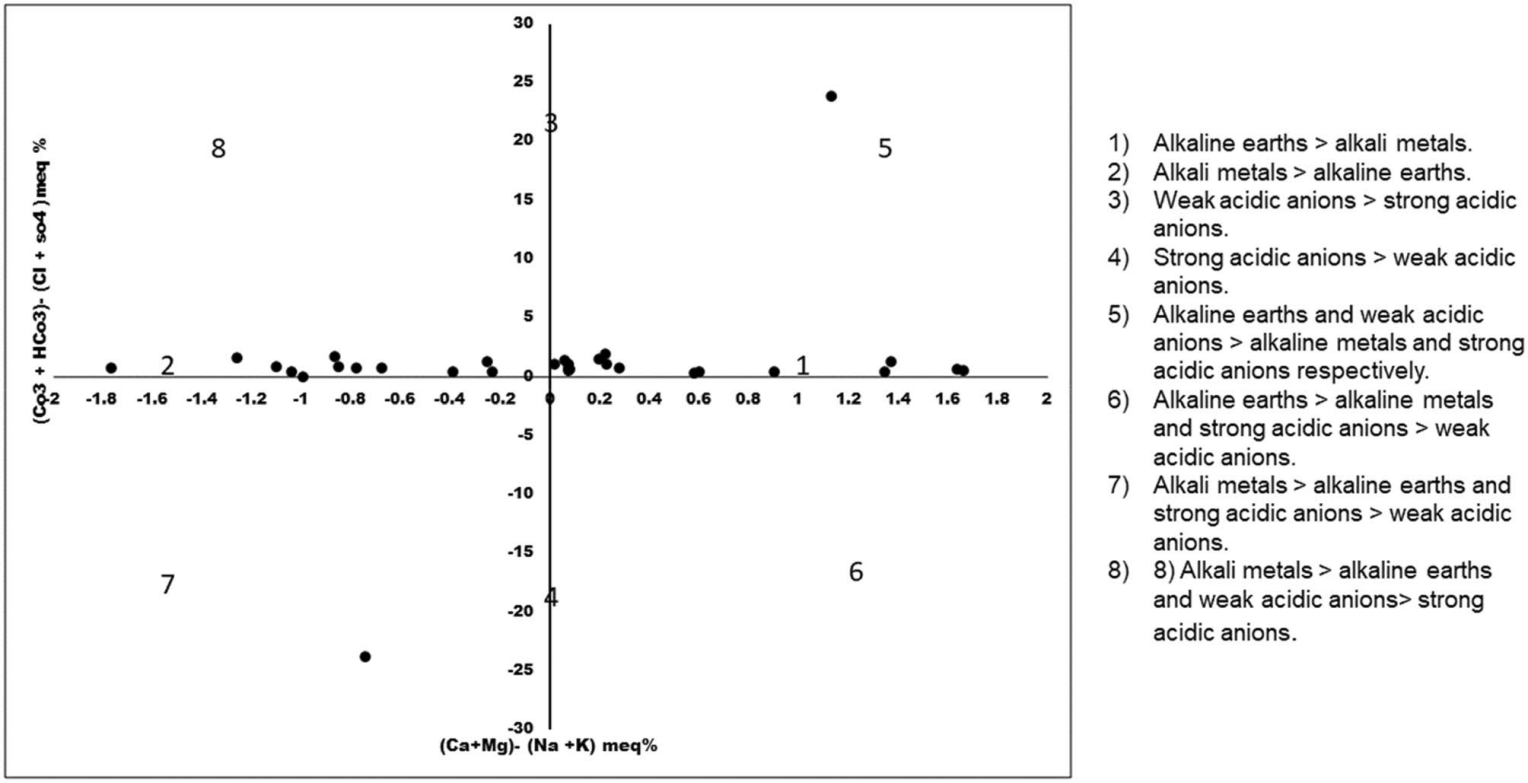
Chadha’s Plot for hydrogeochemical facies.

Figure 10 is the modified version of Durov plot and Piper Plot in which the two equilateral triangles are omitted. In case of Hill Piper plot, the type of water is determined on the data plot when the milliequivalent percentage of major anions and cations are plotted in each triangle irrespective of their triangular field. The central diamond field which provides the overall character of water is the extension of the triangular field. In contrast, the Chadha diagram is plotted between the difference in the alkaline earth (Ca^2+^  + Mg^2+^) and alkali metals (Na^+^  + K^+^) on X axis and the difference in weak acid anions (CO_3_^2-^ + HCO_3_ˉ) and strong acid anions (Clˉ + SO_4_^2^ˉ) on Y axis. Depending on the size of the scale chosen the plot can be square or rectangular field which has all the advantage of diamond- shaped field of Hill- Piper diagram. The rectangular field is divided into eight sub fields to represent the primary character of water. The eight sub fields are as follows: (1) Alkaline earths > alkali metals. (2) Alkali metals > alkaline earths. (3) Weak acidic anions > strong acidic anions. (4) Strong acidic anions > weak acidic anions. (5) Alkaline earths and weak acidic anions > alkaline metals and strong acidic anions respectively. This type of water has temporary hardness and in Chadha’s plot the position of the data points represents Ca^2+^–Mg^2+-^HCO_3_ˉ-type, HCO_3_ˉ dominant Ca^2+^–Mg^2+^-type or Ca^2+^–Mg^2+^ dominant HCO_3_ˉ -type waters. (6)Alkaline earths > alkali metals and strong acidic anions > weak acidic anions. This type of water has permanent hardness and during irrigation usage it does not deposit residual carbonate. The datapoints in the Chadha’s plot represents Ca^2+^–Mg^2+^-dominant Clˉ-type, Ca^2+^–Mg^2+^–Clˉ-type or Clˉ-dominant Ca^2+^–Mg^2+^-dominant Clˉ-type waters. (7) Alkali metals > alkaline earths and strong acidic anions > weak acidic anions. This type of water creates salinity problem and the data points in the Chadha’s plot represents Clˉ -dominant Na^+^-type, Na^+^-dominant Clˉ-type, Na^+^–Clˉ-type, or Na_2_SO_4_-type waters. (8) Alkali metals > alkaline earths and weak acidic anions > strong acidic anions. Residual sodium carbonate deposit and foaming problem occurs in such type of waters. The data points in the Chadha’s plot represents HCO_3_ˉ-dominant Na^+^-type, Na^+^–HCO_3_ˉ-type or Na^+^-dominant HCO_3_ˉ-type waters^54,55^. In present study, the points in the Chadha’s plots lies in different fields (1 and 2) and showing Clˉ -dominant Na^+^ -type, Na^+^-dominant Clˉ-type, Na^+^–Clˉ-type, Na_2_SO_4_–Ca^2+^–Mg^2+^–HCO_3_ˉ-type, HCO_3_ˉ dominant Ca^2+^–Mg^2+^-type or Ca^2+^–Mg^2+^ dominant HCO_3_ˉ-type waters. Most of the samples indicated nature of surface water in the Tawang area as hard (TH > 75) and influence permanent and temporary hardness^47^. The data points set in some location near field 5 and 7 suggested that alkaline earths and weak acidic anions exceedsalkali metals and strong acidic anions zone.

**Figure 11:**
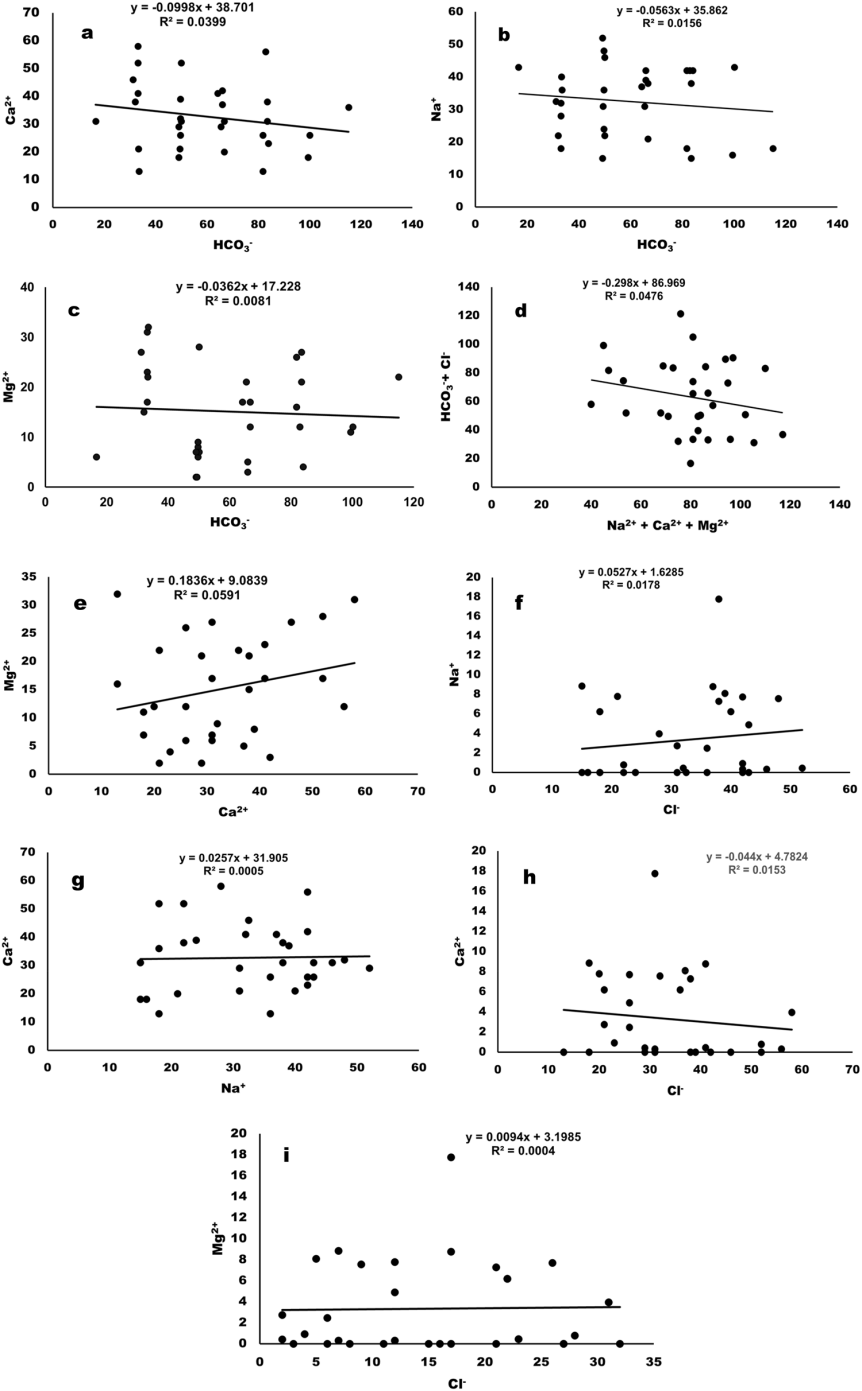
Hydrologic processes, statistical analyses of the correlations between (**a**) Ca^2+^ vs HCO_3_^-^ (**b**) Na^2+^ vs. HCO_3_^-^ (**c**) Mg^2+^ vs. HCO_3_^-^ (**d**) HCO_3_^-^ + Cl^-^ vs. Na^+^  + Mg^2+^  + Ca^2+^ (**e**) Mg^2+^ vs. Ca^2+^ (**f**) Na^2+^ vs. Cl^-^ (**g**) Ca^2+^ vs. Na^2+^ (**h**) Ca^2+^ vs. Cl^-^ & (**i**) Ma^2+^ vs. Cl^-^.

Figure 11a–i shows the statistical analysis of certain pairs of parameters to elucidate the hydrologic processes. The concentration of Ca^2+^ (R^2^ = 0.0399), Na^+^ (R^2^ = 0.0159)and Mg^2+^(R^2^ = 0.0081) in surface water have shown weak association with HCO_3_ˉ concentration. Similarly, HCO_3_ˉ + Clˉ have weak association with Na^+^  + Mg^2+^  + Ca^2+^ ions in the surface water (R^2^ = 0. 0476). However, Mg^2+^, Ca^2+^ and Na^+^ have also showed the weak association amongst them (Mg^2+^ vs Ca^2+^, R^2^ = 0. 0591 and Ca^2+^ vs Na^2+^, R^2^ = 0. 0005). Additionally, the weak association between the cations in the surface water with Clˉ was observed. Similar to this work surface water and groundwater of river Munda Basin was studied and the results are in line with the present study^56^.

**Figure 12:**
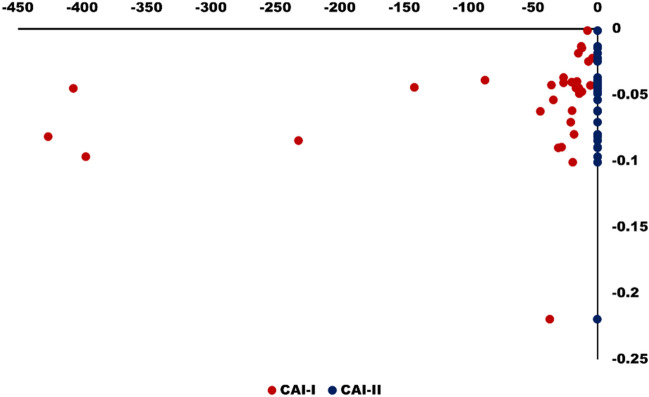
Scatter diagram shows the variation of chloro- alkaline indices of surface water samples in the study area.

Schoeller’s index is also used to study ion exchange that occurs between the surface water and the host environment. To interpret the ion exchange behavior between the surfacewater and the host environment Eqs. (5) and (6) are used. This process is also known as chloro-alkaline indices and all the ions are expressed in meq/L. Depending on the Na^+^ and K^+^ exchange from water with Mg^2+^ and Ca^2+^ in rock/soil, or vice versa, CAI-I and CAI-II values may be positive or negative. If the Chloro-alkaline indices value is positive, it means Na^+^ and K^+^ exchange occurs in water with Mg^2+^ and Ca^2+^ while if it yields negative value this means ion exchange occurs between in Mg^2+^ and Ca^2+^ surface water and Na^+^ and K^+^ in rock/soil^57–59^. In the present study as shown in Fig. 12, all the samples (100%) have generated negative values which revealed a presence of reverse ion exchange controlling surface water chemistry as well as rock-water interaction.

5 $$CAI-I= \frac{{Cl}^{-}-({Na}^{+}+{K}^{+})}{{Cl}^{-}}$$

6 $$CAI-II=\frac{{Cl}^{-}{ - (Na}^{+}+ {K}^{+})}{{SO}_{4}^{2-} +{HCO}_{3}^{-}+{CO}_{3}^{2-}+ {NO}_{3}^{-}}$$

### Process controlling the surface water chemistry

The dissolution of different parent materials yield different ion combinations, so the geological formation occurring within the study area can be assigned to the process that influence the surface water in the Tawang area aquifer. Na^+^ and K^+^ are originating from silicate weathering while Ca^2+^ and Mg^2+^ are from carbonate weathering before evaporation. Additionally, SO_4_^2-^ and Clˉ originate from evaporation while HCO_3_ˉ from carbonate silicates. Studies have been done by many researchers which prove that in water system calcite, dolomite, anhydrite and gypsum weathering and dissolution are very predominant processess.Anthropogenic activity to some extent also generates these parameters. The physiochemical characteristics are also affected by the dissolution of evaporites^47,60^.

**Figure 13:**
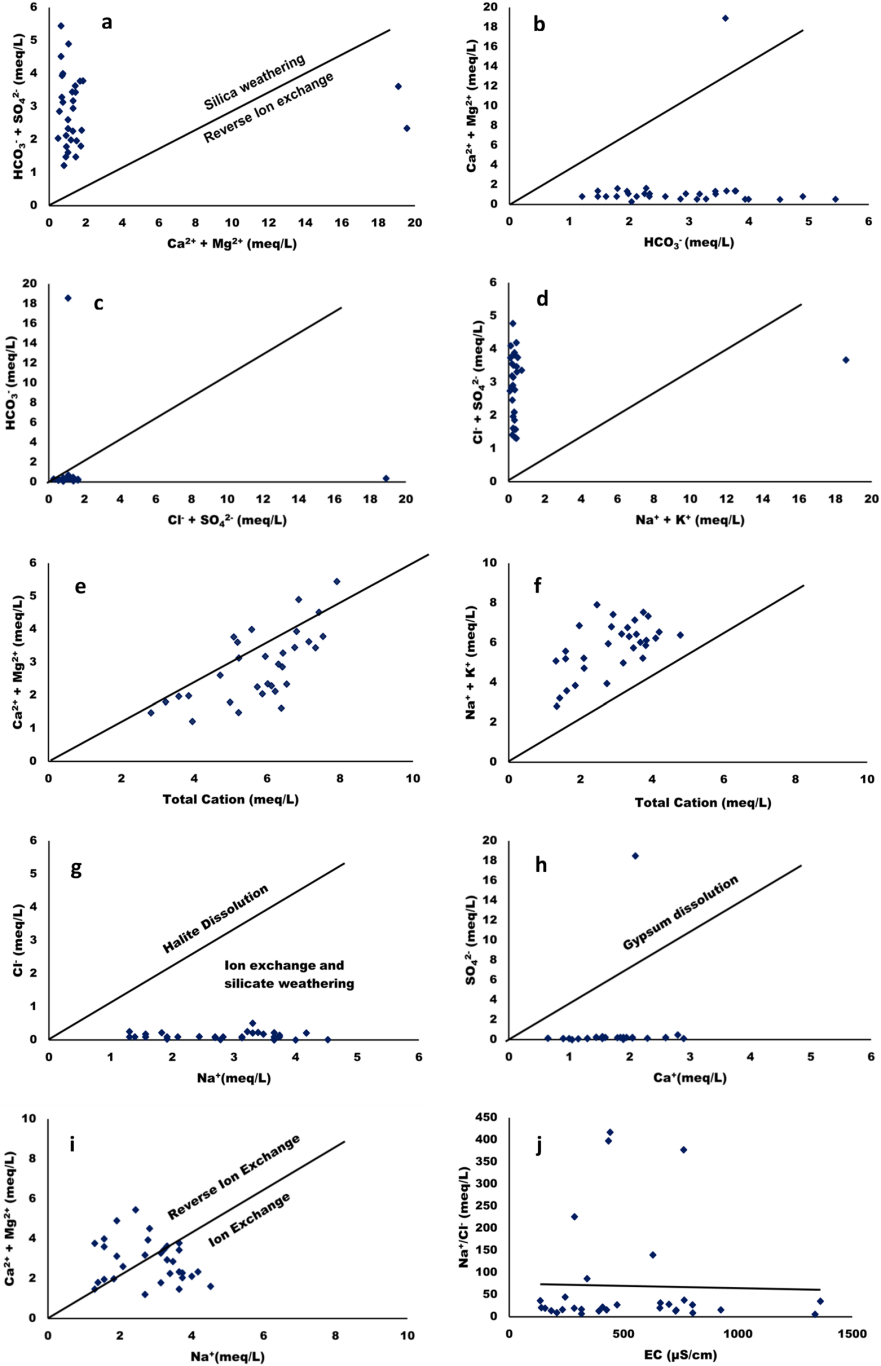
Scatter plots indicating sources of different parameters in surface water of Tawang area.

The scatter plots were used to understand the source of major ions and ion exchange process affecting the surfacewater of Tawang area. These plots compared the different parameters in equivalent concentration (meq/L)^60^. Figure 13a shows the scatter plot of (Ca^2+^  + Mg^2+^) versus (HCO_3_ˉ + SO_4_^2^ˉ) which results in close to 1:1 equiline and highlighted that the water samples from different locations of Tawang has calcite, gypsum dissolution, anhydrite, and dolomite. Meanwhile, reverse ion exchange and carbonate weathering is demonstrated by the abundant amount of Ca^2+^ + Mg^2+^ while silicate weathering can be suggested by the presence of (HCO_3_ˉ + SO_4_^2^ˉ) over (Ca^2+^ + Mg^2+^). This result revealed that all samples show dominance (HCO_3_ˉ + SO_4_^2^ˉ) over (Ca^2+^ + Mg^2+^) indicating that silicate weathering is a reaction that affect the chemistry of the surface water samples in different location of Tawang. Furthermore, in two locationsthe reverse ion exchange is occurring due to excessive (Ca^2+^ + Mg^2+^) over (HCO_3_^-^ + SO_4_^2-^)^52^.

Meanwhile Fig. 13b shows that the plot for (Ca^2+^ + Mg^2+^) vs HCO_3_ˉrepresents that the water samples have fallen below the 1:1 equiline which suggested that in all the locations silicate weathering impacts waterdominantly.If these points fall on or above the divider line, it indicates that HCO_3_ˉ in the surface water is controlled by alkaline earth metals as well as carbonate lithology^47,52,56^. Moreover, (SO_4_^2^ˉ + Cl^-^) vs HCO_3_ˉscattered plot is shown in Fig. 13c in which SO_4_^2-^ + Cl^-^ has shown dominance over HCO_3_^-^ at lower concentration range. Similarly, in Fig. 13d a scattered plot was plotted between (SO_4_^2^ˉ + Clˉ) vs (Na^+^ + K^+^) and it was found that the water sample points are above 1:1 line^55^.

The points plotted againstalkaline earth metals vs total cations in Fig. 13e have fallen above and below the equiline but the maximum number of locations of water samples are below 1:1 line. This might be due to the presence of higher amount of Na^+^ + K^+^ with more dissolved solids. The high (Na^+^ + K^+^)/total cation (50%) and low (Ca^2+^ + Mg^2+^)/(Na^+^  + K^+^) (9.9%) means water chemical composition is greatly affected by silicate weathering along with less influence of carbonate dissolution. Additionally, if the soil is more influenced with alkalinity this could result in more (SO_4_^2^ˉ + Clˉ) and from these findings it can be concluded that the soil source might be having Na^2+^SO_4_^2^ˉ and K^2+^SO_4_^2^ˉ. Figure 13f shows the scattered plot of (Na^+^  + K^+^) vs total cation and the higher ratio of (Na^+^  + K^+^) present in the surface water due to silicate weathering or alkaline soil^47,56^. Furthermore, the Fig. 13g shows the points between Clˉversus Na^+^ to assess the impact of halite dissolution towards the surface water chemistry, whereby all the samples have fallen below the 1:1 equiline. Thus, it can be concluded that the source of Na^+^ is silicate weathering which may be from Na-plagioclaseas mentioned in previous studies^61^.

Another primary process of salination that occurs in the surface water system is depicted using SO_4_^2^ˉvs. Ca^2+^ plot as shown in Fig. 13h. In this plot the points are below 1:1 line which suggest that the gypsum dissolution is less in water samples. A positive relation of (Ca^2+^ + Mg^2+^) vs. Na^+^ was observed in Fig. 13i and it is used to assess the impact of ion exchange on surface water chemistry. In the present study the points are present on both sides of the 1:1 equiline indicative of both ion exchange and reverse ion exchange to occur in water. If the (Na^+^/Clˉ) vs EC (µS/cm) plot will yield a horizontal line,then it has been suggested that the evaporation process is occurring in the water system. If the Na^+^/Clˉ is nearly equal to 1, sodium will be a product of halite dissolution whereas Na^+^/Clˉ > 1 indicative of ions emitted from silicate minerals weathering. In the present work the Na^+^/Clˉmolar ratio is in the range of 5.2–417.2 as shown in Fig. 13j which is greater than 1, indicative of silicate weathering as an indicative of Na^+^ release to the surfacewater^61^.

### Water suitability forirrigation purpose

Water quality must be monitored to maintain soil fertility and better crop output. The low quality water shows harmful impact on heavy clayed soil but can be used for irrigation of sandy and permeable soil through which chemical may pass deep down^47^. To know the suitability of water for irrigation certain parameters like Magnesium hazard (MH), Total hardness (TH), Permeability index (PI), Kelly Index (KI), Sodium adsorption rate (SAR), Sodium percentage (Na%) and Residual sodium carbonate (RSC) are used.

**Figure 14:**
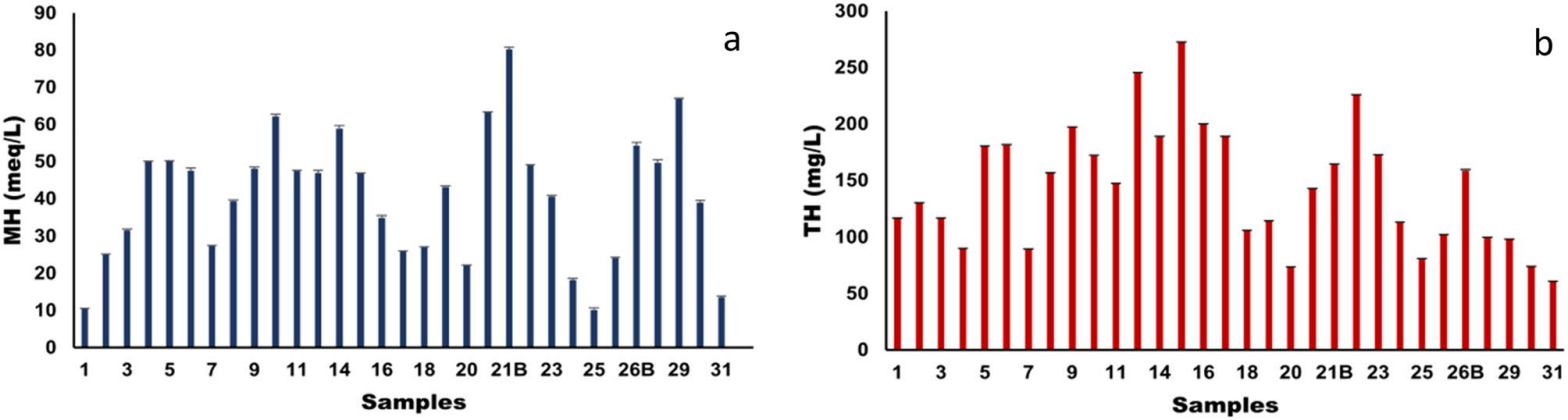
(**a**) Magnesium hazard and (**b**) Total hardness of the water samples collected from different locations of Tawang.

Szabolcs and Darab proposed a formula as mentioned in Table 8 for the calculation of Magnesium hazard (MH) in meq/L. According to this formula if MH < 50 meq/L then the water is suitable for irrigation while if MH > 50 meq/L then the water is unsuitable for irrigation^62^. In the present study as shown in Fig. 14a, 80% of water samples from different locations have MH scores within the safe limit of 50 meq/L indicating suitability for irrigation. While 20% of water samples have MH score > 50 meq/L which means these water samples have more Mg^2+^ over Ca^2+^which adversely affect the soil quality leading to poor irrigation yield. In most of the surface water the state of equilibrium is maintained by the Ca^2+^ and Mg^2+^ions,however if the in-equilibrium occurs between these two ions the soil will become alkaline due to high Mg^2+^ concentration in water and subsequently leads to reduction in crop yield. Additionally, in highly saline and predominantly sodium dense water, Mg^2+^ ions will negatively impact soil structure.

**Table 8:** Irrigation water classification Parameters.

S. No	Classification pattern	Formula	Ranges	Categories	% of samples
1	Magnesium Hazard (MH) (Raghunath 1987)	$MH =\frac{{Mg}^{2+}}{{Ca}^{2+}+ {Mg}^{2+}}*100$	< 50 > 50	Suitable Unsuitable	80 20
2	Total Hardness	$TH =2.497{Ca}^{2+}+ 4.11{Mg}^{2+}$	0–75 75–150 150–300 > 300	Soft Moderately hard Hard Very hard	03 14 14 0
3	Permeability Index (PI) (Doneen 1964)	$PI= \frac{{(Na}^{+}+ \sqrt{{HCO}_{3}^{-}})}{{(Ca}^{2+}+{Mg}^{2+}+{Na}^{+})}*100$	< 75 > 75	Suitable Unsuitable	93 7
4	Kelly’s Index	$KI=\frac{{Na}^{+}}{{(Ca}^{2+}+{Mg}^{2+ })}$	< 1 ≥ 1	Suitable Unsuitable	58 42
5	Sodium absorption ratio (SAR) (Richard 1954)	$SAR = \frac{{Na^{ + } }}{{\sqrt {Ca^{{2 + }} + Mg^{{2 + }} /2} }}$	0–10 10–18 18–26 ≥ 26	Low Medium High Very high	100 0 0 0
6	Percent sodium (% Na) (Wilcox 1955)	$Na\%= \frac{{(Na}^{+}+ {K}^{+})*100}{{Ca}^{2+}+ {Mg}^{2+}+{Na}^{+}+ {K}^{+}}$	0–20 20–40 40–60 60–80 > 80	Excellent Good Permissible Doubtful Unsuitable	0 20 48 32 0
7	Electrical conductivity (EC) (Wilcox 1955)	NA	< 250 250–750 750–2250 2250–5000 > 5000	Excellent Good Permissible Doubtful Unsuitable	23 55 22 0 0
8	RSC	$RSC=\left({CO}_{3}^{2-}+ {HCO}_{3}^{-}\right)-({Ca}^{2+}+ {Mg}^{2+})$	< 1.25 1.25–2.5 ≥ 2.5	Safe Fair quality Unsuitable	99 0 1

Due to precipitated Ca^2+^ and Mg^2+^ ions in water, the quality of permanent and temporary hardness can be deduced from action of soap on it. Calcium carbonate is the reason for temporary hardness in water which can be removed byheating,whilethepermanent hardness is due to the presence of both Ca^2+^ and Mg^2+^ ions andcan be removed by ion exchange. In the present study, the total hardness (TH) was calculated as per Toddmentioned in Table 8 and is expressed in mg/L. The total hardness in this study ranges from 73.75 ± 0.5 to 272.39 ± 0.08 mg/L as shown in Fig. 14b whereby its optimal limit is 80–100 mg CaCO_3_/L.

**Figure 15:**
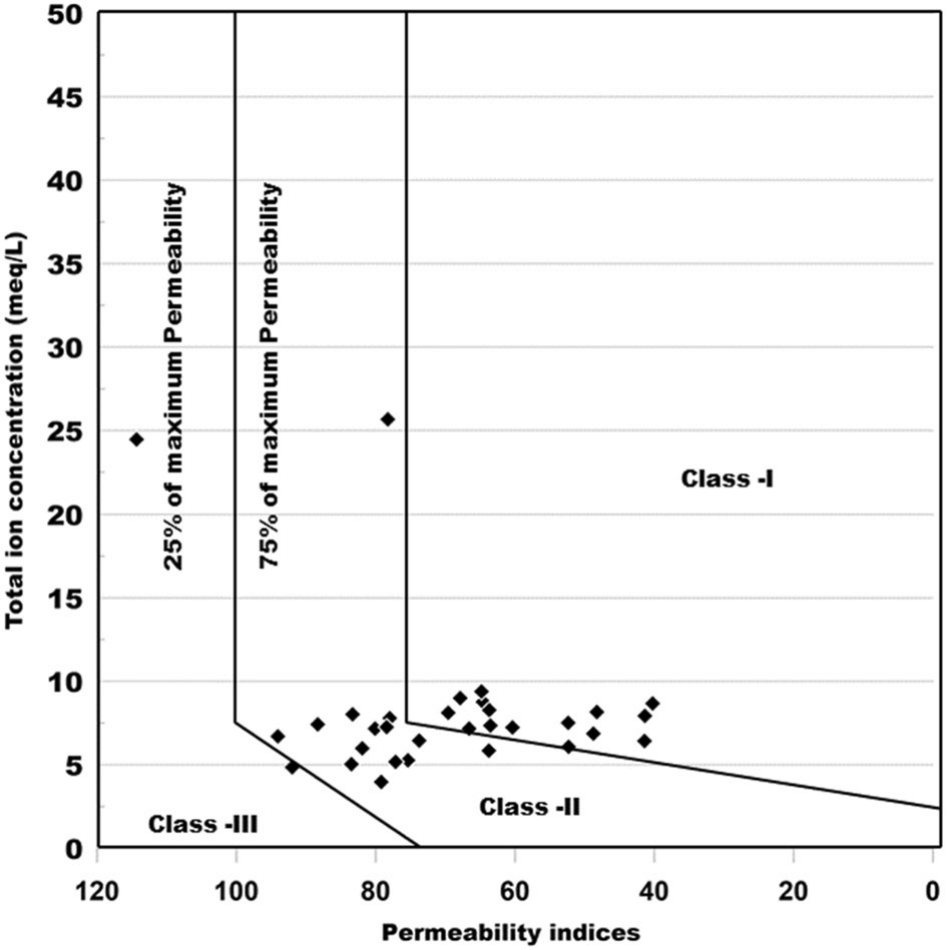
Irrigation water classification based on Permeability index.

The long-term use of Na^+^, Ca^2+^, Mg^2+^ and HCO_3_ˉ rich water affects the soil permeability. To find out the suitability of water Doneen used the Permeability index (PI) and classified irrigation water in three classes which are Class-I, Class-II and Class-III. Figure 15 (Grapher16.3.410, graphersupport@goldensoftware.com) shows the classification of irrigation water based on permeability index calculated as per the formula mentioned in Table 8. According to this classification only Class-I and Class-II types of water are suitable for irrigation due to 75% or more maximum permeability score while Class-III is not suitable for irrigation due to 25% maximum permeability. In the present research work, 48% of the samples falls in Class-I and 45% of the water samples falls in Class-II, indicating that the water is good for irrigation purpose. However, 7% of water sample falls into Class-III, indicating that water is not suitable for irrigation.

Kelly index (KI)is also another useful method to classify water for irrigation. It can be calculated using the following formula as mentioned in Table 8. According to this index, KI < 1.0 is indicative of good water for irrigation while KI > 1.0 is indicative of bad water and unsuitable for irrigation^47,52^. In the present study the 58% of water samples obtained from different location of Tawang has yielded KI values < 1.0 while 42% of them yielded KI value > 1.0. Hence, 58% of water samples from different location is suitable for irrigation.

**Figure 16:**
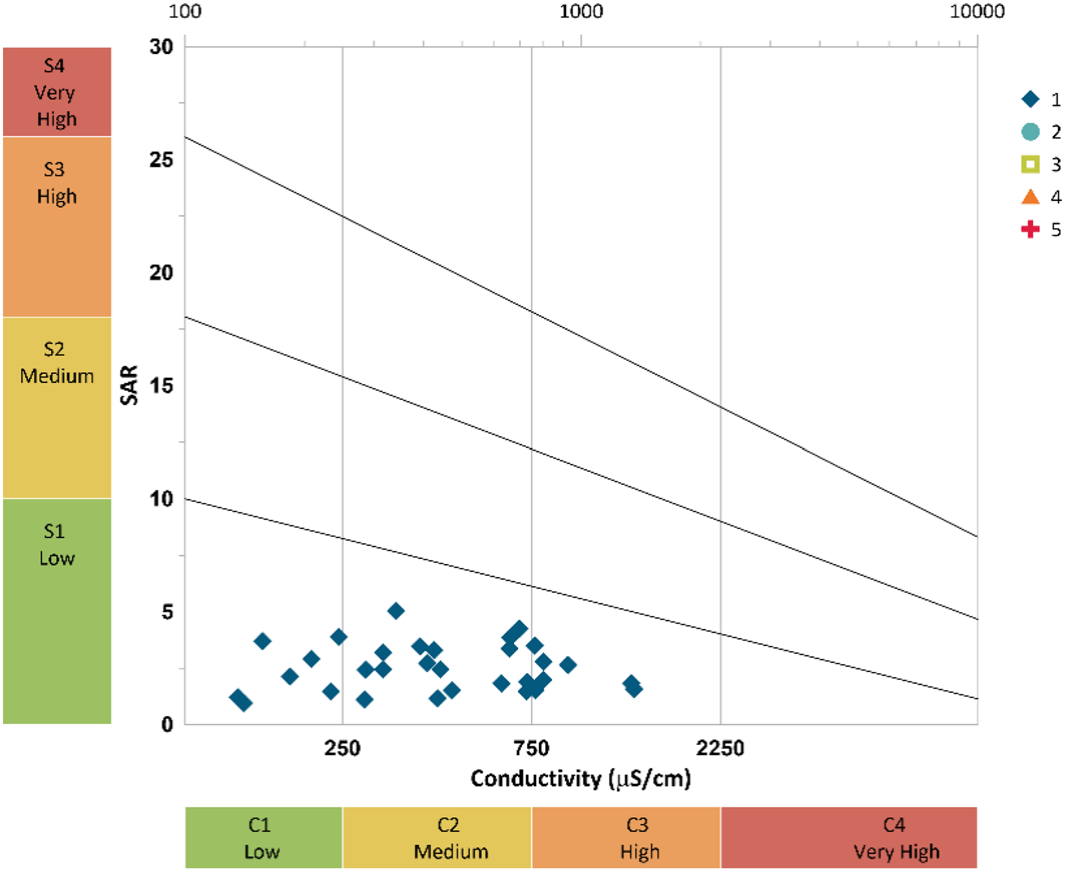
Classification of irrigation water using Wilcox diagram.

Sodium adsorption rate (SAR) is a parameter to calculate the correlation between soluble divalent cations (Ca^2+^ and Mg^2+^) and Na^+^. If SAR value is higher, it means the Na^+^ concentration is higher with respect to Ca^2+^ and Mg^2+^. An alkaline soil is developed from high Na^+^ concentration and reduced soil permeability. The SAR is determined by using formula mentioned in Table 8 and based on this the irrigation water is classified into four alkali categories: S1: low (0–10), S2: medium (10–18), S3: high (18–26) and S4: very high (> 26)^63^. In the present study as shown in Fig. 16 (Grapher 16.3.410, graphersupport@goldensoftware.com), the SAR values in the study area ranges from 0.95 to 5.03 and as per Richard’s classification all the samples fall into the low sodium hazard and low to very high salinity hazard. These results support the findings of KI. Therefore, it can be concluded that the maximum water samples from different location of Tawangare suitable for irrigation purpose.

**Figure 17:**
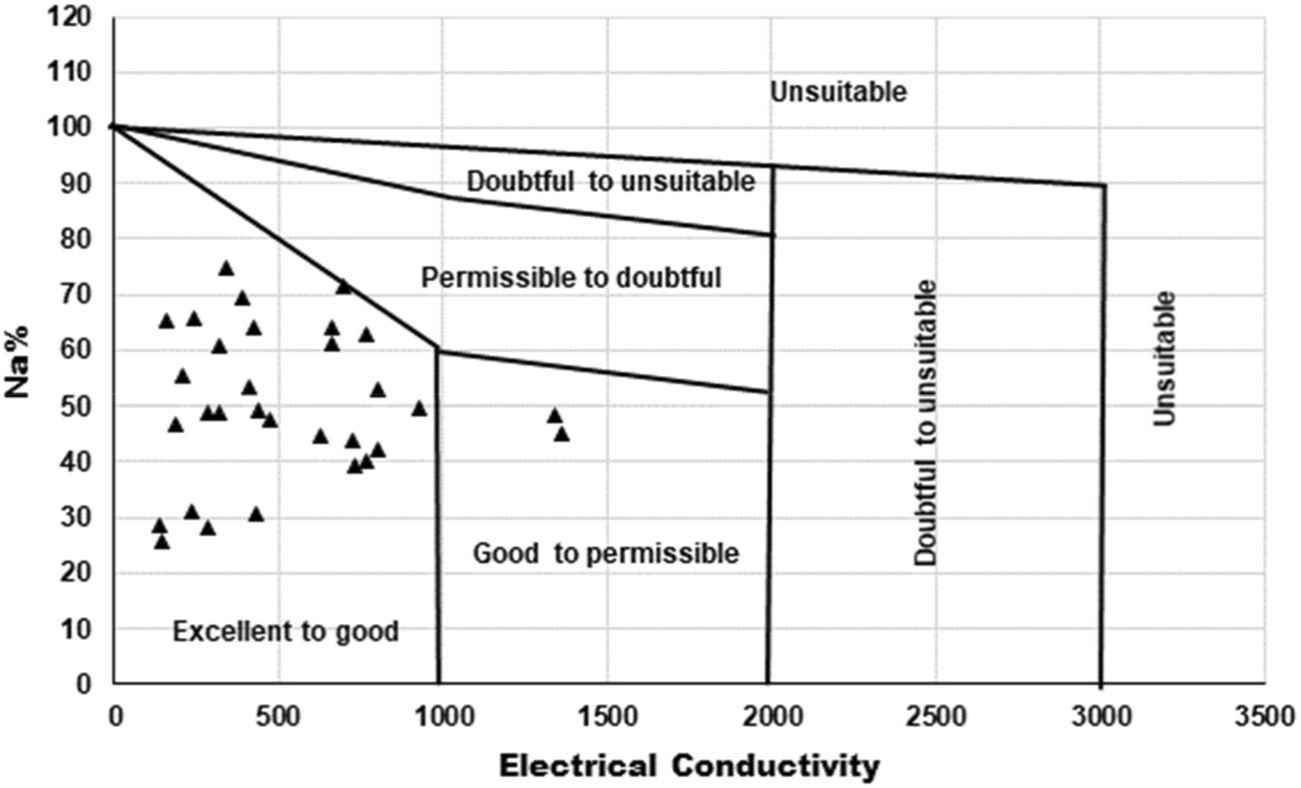
Surface water rating using Sodium Percentage and Electrical Conductivity.

Sodium is deemed as an important cation in water irrigation due to its ability to reduce soil fertility, as high cation concentration shows negative effect on plant growth. The sodium percentage in this study was also calculated by using Wilcox formula as mentioned in Table 8^64.^ According to BIS the maximum acceptable limit of Na% is 60% and above that limit it is harmful for growth of plants. Additionally, the higher concentration of sodium increases the hardness in soil leading to reduction in soil permeability^47^. Hence, based on the Table 8, 20% of the samples lie in good category, 48% in permissible while 32% in doubtful in terms of their suitability for irrigation. Additionally, a graph has been plotted between Na% and EC as per Wilcox to determine the water suitability for irrigation and from Fig. 17 it was found that 94% of samples are excellent to good for agricultural usages. The remaining 6% of the samples lies in good to permissible category and can be used for irrigation purpose. From these results it can be concluded that for irrigation purpose water should have high concentration of Ca^2+^ and Mg^2+^ ions and lesser Na^+^.

The residual sodium carbonates (RSC) are another method to check water irrigation property. RSC can be calculated using the difference in sum of carbonate and bicarbonate to the sum of calcium and magnesium in water as mentioned in Table 8. The excess amount of carbonate and bicarbonate altered the soil physical property either by increasing its salinity or itself get precipitated leading to decrease in soil fertility. The high concentration of bicarbonate precipitate Ca^2+^ and Mg^2+^ in water and reduce their amount that ultimately leads tohigh concentration of CO_3_^2-^ and HCO_3_ˉ in the solution expressed as HCO_3_ˉ hazard. RSC is also used to assess the relationship between alkaline earths with weak acids to assess the water quality for irrigation. If weak acid > alkaline earths, then soil permeability is damaged, as alkaline earths become precipitated in the soil. Additionally, excessive carbonate and bicarbonate in water also intrude alkaline earth over the permissible limit and finally affect the agricultural crop. The RSC value is categorized into safe for irrigation (< 1.25 epm), fair quality water (1.25–2.5 epm) and unsuitable for irrigation (> 2.5 epm)^47,52,56^.In this study, 99% of the surface water samples have been categorized as safe for irrigation while the remaining 1% unsuitable for irrigation. The negative RSC values indicates that the calcium and magnesium are partially precipitated.

## Conclusion

In the present study, the water quality of 31 different locations of Tawangduring the winter season were evaluated from the viewpoint of its suitability for drinking and irrigation. For calculation of water quality, 15 parameters were selected represented by pH, TDS, anions, cations, EC, salinity, turbidity for PCA analysis. Different statistical toolssuch as PCA and coorelation analysis were used in this paper to derive the relationship between different parameters of water as mentioned below.

1. In the present study, the average WQI value is found to be 82.49. The WQI results of 61% samples are in the range of 0–50 which are considered as good for drinking, while 39% are unsuitable for drinking showing value > 50. In terms of WQI, the Tawang water samples from most of the sites have good water quality except some which show poor water quality.
2. Hill piper plot showed that the alkaline earth dominates alkali metal and weak acid exceeds strong acid. This plot also showed that the Ca^2+^–HCO_3_ˉ, Na^+^–HCO_3_ˉ and Na^+^–Clˉ type of water is dominant in this area.Gibbs plot also revealed that rock weathering is the main process which controls the surface water The dominance due to precipitation was also observed, which may be due to continuous outflow of surface water thus having short rock-water interaction. The silicate weathering is found to be dominant process rather than carbonate weathering due to rich silicate minerals lithology. For increasing concentration of alkali metals in the surface water of Tawang, reverse ion exchange process has played an active role. Both Na^+^ and Ca^2+^ are observed as dominant cations while HCO_3_ˉ and SO_4_^2^ˉ as dominant anions. The values in the chloro-alkaline indices in this study were negative indicating the exchange of Ca^2+^ and Mg^2+^ ions by Na^+^ and K^+^ ions of rock material.
3. The scatter plot of Ca^2+^ + Mg^2+^ vs total cation and Na^+^ + K^+^ vs total cation has specified the silicate weathering as a dominant source of major ions. Furthermore, the scattered plot of (Ca^2+^ + Mg^2+^) vs (HCO_3_ˉ + SO_4_^2^ˉ), (Ca^2+^ + Mg^2+^) vs HCO_3_ˉ and (SO_4_^2^ˉ + Clˉ) vs HCO_3_ˉindicated that both silicate weathering and reverse ion exchange processes have played an important role in geochemical reactions.
4. The parameters like MH, KI, PI, SAR, Na% and RSC have shown that the water from different locations of Tawang is suitable for irrigation. Some water samples were found to be hard with permanent and temporary hardness. However 93% of the samples have shown PI score < 75, which indicates the suitability of the samples for irrigation.

Hence this study has thrown some light on the water quality of untouched surface water sources of theTawang area. The study has revealed the suitability of most of the water samples for human consumption as well as irrigation. From the detailed water quality analysis and geochemical characteristics it can be inferred that those surface water sources lying at an altitude of maximum 4465 m in the studied area have not been exposed to any kind of anthropogenic activities. Hence the obtained results are in coorelation with natural activities only, which make it even more valuabledatabase asset for future references.

## References

[1] Mukate S (2019). Development of new integrated water quality index (IWQI) model to evaluate the drinking suitability of water. Ecol. Ind..

[2] Wu Z (2018). Assessing river water quality using water quality index in Lake Taihu Basin, China. Sci. Total Environ..

[3] Uddin MG, Nash S, Olbert AI (2021). A review of water quality index models and their use for assessing surface water quality. Ecol. Ind..

[4] Noori R (2019). A critical review on the application of the National sanitation foundation water quality index. Environ. Pollut..

[5] Kumar R (2019). Hydro-geochemical characteristics of glacial meltwater from Naradu Glacier catchment, Western Himalaya. Environmental Earth Sciences.

[6] Duraisamy S (2019). Hydrogeochemical characterization and evaluation of groundwater quality in Kangayam taluk, Tirupur district, Tamil Nadu, India, using GIS techniques. Environ. Geochem. Health.

[7] Adimalla N, Taloor AK (2020). Hydrogeochemical investigation of groundwater quality in the hard rock terrain of South India using Geographic Information System (GIS) and groundwater quality index (GWQI) techniques. Groundw. Sustain. Dev..

[8] Şener Ş, Şener E, Davraz A (2017). Evaluation of water quality using water quality index (WQI) method and GIS in Aksu River (SW-Turkey). Sci. Total Environ..

[9] Tripathi M, Singal SK (2019). Use of Principal Component Analysis for parameter selection for development of a novel Water Quality Index: A case study of river Ganga India. Ecol. Ind..

[10] Tian Y (2019). Using a water quality index to assess the water quality of the upper and middle streams of the Luanhe River, northern China. Sci. Total Environ..

[11] Ewaid SH (2020). Development and evaluation of a water quality index for the Iraqi Rivers. Hydrology.

[12] Ram A (2021). Groundwater quality assessment using water quality index (WQI) under GIS framework. Appl. Water Sci..

[13] Abba SI (2020). Emerging evolutionary algorithm integrated with kernel principal component analysis for modeling the performance of a water treatment plant. J. Water Process Eng..

[14] Taloor AK (2020). Spring water quality and discharge assessment in the Basantar watershed of Jammu Himalaya using geographic information system (GIS) and water quality Index (WQI). Groundw. Sustain. Dev..

[15] Kawo NS, Karuppannan S (2018). Groundwater quality assessment using water quality index and GIS technique in Modjo River Basin, central Ethiopia. J. Afr. Earth Sc..

[16] Zanotti C (2019). Groundwater and surface water quality characterization through positive matrix factorization combined with GIS approach. Water Res..

[17] Rice, E.W., Baird, R.B. & Eaton A.D. In *Standard Methods for the Examination of Water and Wastewater*, 23rd edn (American Public Health Association, American Water Works Association, Water Environment Federation, 2017)

[18] Standard, I., *Drinking water-specification.* 1st Revision, IS, 1991. **10500**.

[19] Cotruvo JA (2017). 2017 WHO guidelines for drinking water quality: First addendum to the fourth edition. J. Am. Water Works Assoc..

[20] Federation, W.E. and A. Association, *Standard methods for the examination of water and wastewater.* American Public Health Association (APHA): Washington, DC, USA, 2005. **21**.

[21] Ewaid SH, Abed SA, Kadhum SA (2018). Predicting the Tigris River water quality within Baghdad, Iraq by using water quality index and regression analysis. Environ. Technol. Innov..

[22] Mahapatra SS (2012). Prediction of water quality using principal component analysis. Water Qual. Expo. Health.

[23] Tokatli C (2019). Drinking water quality assessment of Ergene River Basin (Turkey) by water quality index: Essential and toxic elements. Sains Malay..

[24] Călmuc, V.A., *et al*., Various methods for calculating the water quality index. *Analele Universității” Dunărea de Jos” din Galați. Fascicula II, Matematică, fizică, mecanică teoretică/Annals of the” Dunarea de Jos” University of Galati. Fascicle II, Mathematics, Physics, Theoretical Mechanics*, 2018. **41**(2): p. 171–178.

[25] Anyanwu, E.D. and C.S. Emeka, Application of water quality index in the drinking water quality assessment of a southeastern Nigeria river. *Food Environ. Saf. J.,* **18**(4), 308–314. (2020).

[26] Ustaoğlu F, Tepe Y, Taş B (2020). Assessment of stream quality and health risk in a subtropical Turkey river system: A combined approach using statistical analysis and water quality index. Ecol. Ind..

[27] Menberu Z, Mogesse B, Reddythota D (2021). Evaluation of water quality and eutrophication status of Hawassa Lake based on different water quality indices. Appl. Water Sci..

[28] Khatri N (2020). Analysis and assessment of ground water quality in Satlasana Taluka, Mehsana district, Gujarat, India through application of water quality indices. Groundw. Sustain. Dev..

[29] BIS, I.S.D.W.S., *Bureau of Indian Standards.* New Delhi, 2012: p. 2–3.

[30] Devesa R, Dietrich A (2018). Guidance for optimizing drinking water taste by adjusting mineralization as measured by total dissolved solids (TDS). Desalination.

[31] Wu T, Brant JA (2020). Magnetic field effects on pH and electrical conductivity: Implications for water and wastewater treatment. Environ. Eng. Sci..

[32] Kumar R (2019). Distribution of trace metal in Shaune Garang catchment: evidence from particles and nanoparticles. Mater. Today: Proc..

[33] Dubey, R., *et al.*, *Survey of natural water sources of Tawang region and studies of their physico-chemical and bacterial contamination of water.* 2020.

[34] Balázs B (2018). Extracting water-related features using reflectance data and principal component analysis of Landsat images. Hydrol. Sci. J..

[35] Shrestha N (2021). Factor analysis as a tool for survey analysis. Am. J. Appl. Math. Stat..

[36] Jang D, Choi G (2017). Estimation of non-revenue water ratio for sustainable management using artificial neural network and Z-score in Incheon, Republic of Korea. Sustainability.

[37] Mukherjee I, Singh UK (2018). Groundwater fluoride contamination, probable release, and containment mechanisms: A review on Indian context. Environ. Geochem. Health.

[38] Zango MS (2019). Hydrogeochemical controls and human health risk assessment of groundwater fluoride and boron in the semi-arid North East region of Ghana. J. Geochem. Explor..

[39] Kumari M, Rai S (2020). Hydrogeochemical evaluation of groundwater quality for drinking and irrigation purposes using water quality index in semi arid region of India. J. Geol. Soc. India.

[40] Ram A (2020). Assessment of groundwater quality using water quality index (WQI) in Kulpahar watershed, District Mahoba, Uttar Pradesh, India. J. Indian Assoc. Environ. Manag. (JIAEM).

[41] Mridha D (2021). Fluoride exposure and its potential health risk assessment in drinking water and staple food in the population from fluoride endemic regions of Bihar, India. Groundw. Sustain. Dev..

[42] Narsimha A, Sudarshan V (2017). Contamination of fluoride in groundwater and its effect on human health: A case study in hard rock aquifers of Siddipet, Telangana State India. Appl. Water Sci..

[43] Nieder R, Benbi DK, Reichl FX (2018). Reactive water-soluble forms of nitrogen and phosphorus and their impacts on environment and human health. Soil components and human health.

[44] Agency, U.E.P., *Drinking water standards and health advisories*. 2018, US Environmental Protection Agency Washington, DC.

[45] Ram A (2021). Groundwater quality assessment using water quality index (WQI) under GIS framework. Appl. Water Sci..

[46] Tanvir Rahman MATM (2017). Groundwater characterization and selection of suitable water type for irrigation in the western region of Bangladesh. Appl. Water Sci..

[47] Gaury PK, Meena NK, Mahajan A (2018). Hydrochemistry and water quality of Rewalsar Lake of Lesser Himalaya, Himachal Pradesh, India. Environ. Monitor. Assess..

[48] Ravikumar P, Somashekar R, Prakash K (2015). A comparative study on usage of Durov and Piper diagrams to interpret hydrochemical processes in groundwater from SRLIS river basin, Karnataka, India. Elixir Earth Sci..

[49] Lloyd, J.W. and Heathcote, J., *Natural inorganic hydrochemistry in relation to ground water.* 1985.

[50] Sharma G (2021). Application of multivariate statistical analysis and water quality index for quality characterization of Parbati River, Northwestern Himalaya, India. Discover. Water.

[51] Gibbs R (1970). Mechanisms controlling world water chemistry. Sci. J..

[52] Kumar P, Mahajan AK, Kumar A (2020). Groundwater geochemical facie: Implications of rock-water interaction at the Chamba city (HP), northwest Himalaya, India. Environ. Sci. Pollut. Res. Int..

[53] Kumar R (2019). Hydro-geochemical analysis of meltwater draining from Bilare Banga glacier, Western Himalaya. Acta Geophys..

[54] Chadha D (1999). A proposed new diagram for geochemical classification of natural waters and interpretation of chemical data. Hydrogeol. J..

[55] Jain C, Sharma S, Singh S (2018). Physico-chemical characteristics and hydrogeological mechanisms in groundwater with special reference to arsenic contamination in Barpeta District, Assam (India). Environ. Monit. Assess..

[56] Shamsuddin MKN (2019). Geochemical characteristic and water quality index of groundwater and surface water at Lower River Muda Basin, Malaysia. Arab. J. Geosci..

[57] Schoeller, H., Geochemistry of groundwater. In* ‘Groundwater Studies–an International Guide for Research and Practice’.(Eds RH Brown, AA Konoplyantsev, J. Ineson, and VS Kovalevsky.) pp. 1–18*. 1977, UNESCO: Paris, France.

[58] Barik R, Pattanayak SK (2019). Assessment of groundwater quality for irrigation of green spaces in the Rourkela city of Odisha, India. Groundw. Sustain. Dev..

[59] Kant N, Singh PK, Kumar B (2018). Hydrogeochemical characterization and groundwater quality of Jamshedpur urban agglomeration in Precambrian terrain, Eastern India. J. Geol. Soc. India.

[60] Kumar P, Kumar P (2019). Removal of cadmium (Cd-II) from aqueous solution using gas industry-based adsorbent. SN Appl. Sci..

[61] Li X (2019). Hydro-geochemistry of the river water in the Jiulongjiang River basin, Southeast China: Implications of anthropogenic inputs and chemical weathering. Int. J. Environ. Res. Public Health.

[62] Szabolcs I, Darab K (1964). Radio-active technique for examining the improving effect of CaCO_3_ on alkali (Szik) soils. Acta Agron. Hung..

[63] McGeorge, W.T. Diagnosis and Improvement of Saline and Alkaline Soils. *Soil Science Society of America Journal*, **18**, 348–348. 10.2136/sssaj1954.03615995001800030032x (1954).

[64] Wilcox, L., *Classification and use of irrigation waters*. 1955: US Department of Agriculture.

